# Concerted ESCRT and clathrin recruitment waves define the timing and morphology of intraluminal vesicle formation

**DOI:** 10.1038/s41467-018-05345-8

**Published:** 2018-07-26

**Authors:** Eva Maria Wenzel, Sebastian Wolfgang Schultz, Kay Oliver Schink, Nina Marie Pedersen, Viola Nähse, Andreas Carlson, Andreas Brech, Harald Stenmark, Camilla Raiborg

**Affiliations:** 1 Department of Molecular Cell Biology, Institute for Cancer Research, Oslo University Hospital, Montebello, N-0379 Oslo, Norway; 2 Centre for Cancer Cell Reprogramming, Institute of Clinical Medicine, Faculty of Medicine, University of Oslo, N-0316 Oslo, Norway; 3 Department of Mathematics, University of Oslo, N-0316 Oslo, Norway

## Abstract

The endosomal sorting complex required for transport (ESCRT) machinery mediates cargo sorting, membrane deformation and membrane scission on the surface of endosomes, generating intraluminal vesicles (ILVs) to degrade signaling receptors. By live-cell imaging of individual endosomes in human cells, we find that ESCRT proteins are recruited in a repetitive pattern: ESCRT-0 and -I show a gradual and linear recruitment and dissociation, whereas ESCRT-III and its regulatory ATPase VPS4 display fast and transient dynamics. Electron microscopy shows that ILVs are formed consecutively, starting immediately after endocytic uptake of cargo proteins and correlating with the repeated ESCRT recruitment waves, unraveling the timing of ILV formation. Clathrin, recruited by ESCRT-0, is required for timely ESCRT-0 dissociation, efficient ILV formation, correct ILV size and cargo degradation. Thus, cargo sorting and ILV formation occur by concerted, coordinated and repetitive recruitment waves of individual ESCRT subcomplexes and are controlled by clathrin.

## Abstract (web summary)

Intraluminal vesicles are formed by the endosomal sorting complex required for transport (ESCRT) machinery. Here, the authors unravel the timing of vesicle budding, and that endosomal clathrin regulates concerted recruitment of ESCRT subcomplexes, required for efficient membrane remodeling.

## Introduction

To limit sustained growth factor stimulation of cells, ligands and receptors enter the endocytic degradative pathway for destruction in the lysosome. After endocytosis of the activated and ubiquitinated receptors, they are isolated from the cytoplasm to terminate signaling via the formation of intraluminal vesicles (ILVs), resulting in multivesicular endosomes (MVEs). This process of receptor sorting, membrane deformation and vesicle scission is mediated by the endosomal sorting complex required for transport (ESCRT) machinery, which consists of four multiprotein subcomplexes, ESCRT-0, -I, -II and -III, and the ATPase VPS4^1^. ESCRT-0 recognizes ubiquitin residues on the cargo and sorts it into spatially restricted areas on the endosome membrane^2^. Cargo sorting is supported by clathrin, which is recruited to endosomes by ESCRT-0 and has been proposed to concentrate the sorting machinery in restricted microdomains^3–5^. Since ESCRT-I and –II can both interact with ubiquitin and form a supercomplex with variable structural conformations, they may be involved in both cargo transfer and the initial membrane deformation^6^. ESCRT-III consists of CHMP6, CHMP4, CHMP3 and CHMP2 proteins, which, upon activation, polymerize into filaments and can adopt a variety of secondary shapes (summarized in^7^). ESCRT-III together with the VPS4 complex is crucial for membrane scission^8^.

The ESCRT machinery not only mediates the formation of MVEs, but is also involved in many other cellular membrane deformation and scission events, such as cytokinetic abscission, virus budding, plasma membrane repair and nuclear envelope reformation and repair (summarized in refs. ^9–11^). All these cellular processes show a similar topology, resulting in a budding event away from the cytoplasm, which is a “reverse-topology” when compared to classical clathrin-mediated endocytosis, where vesicles are formed towards the cytosol. In contrast to clathrin-mediated endocytosis, the mechanism of ESCRT-mediated membrane deformation and scission is still unknown^7^. Likewise, the timing of ILV formation and the dynamics of ESCRT proteins during this process are unknown and may range from seconds to minutes, similar to virus budding^12–15^, or may last about 1 h, as during cytokinetic abscission^16,17^. In addition, while the order of ESCRT-0 to ESCRT-III recruitment has been well characterized by yeast epistasis analysis^18–21^ and mammalian experiments^22–25^, with ESCRT-III being dependent on the earlier ESCRT complexes for its recruitment and membrane association, it remains unclear whether ESCRT-0 and ESCRT-III act simultaneously or sequentially.

In the current study we elucidate the dynamics of the ESCRT machinery on endosomes, the timing of ILV formation and the role of the clathrin coat for the formation of ILVs.

## Results

### “Late” ESCRTs localize to early endocytic compartments

**Fig. 1:**
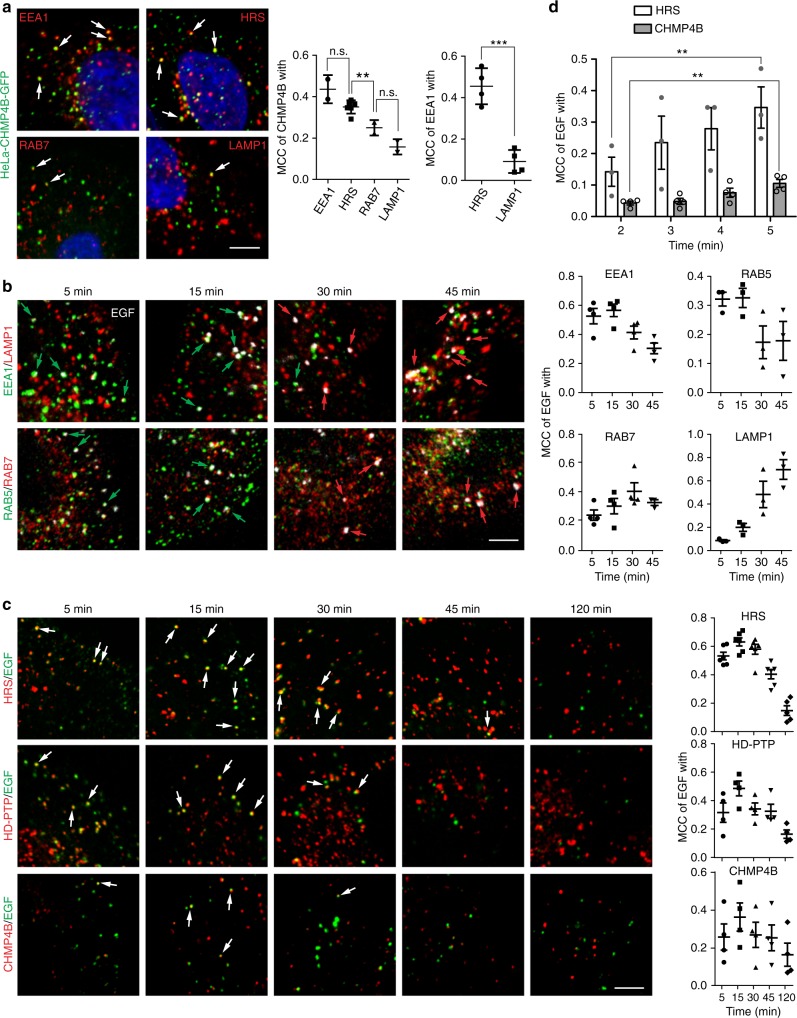
ESCRT proteins localize to “late” early endocytic compartments. **a** Colocalization analysis of unstimulated fixed HeLa cells stably expressing CHMP4B-GFP shows good overlap of CHMP4B with early endocytic compartments. Manders’ colocalization coefficient (MCC): overlap of CHMP4B with endocytic markers and overlap of EEA1 with early or late endocytic markers as positive and negative control for colocalization. Statistics CHMP4B: One-way ANOVA, *p* = 0.0004. Tukey’s post hoc test; **p < 0.01; n.s. not statistically significant; Statistics EEA1: *t*-test, ***p < 0.001; *n* = 2–6 confocal experiments with 3-4 images (each 4–5 cells) per condition. Shown are details. Arrows: CHMP4B-GFP on endosomes. Data are mean ± SD. **b** Pulse-chase experiment: HeLa cells were stimulated for 2 min with 50 ng ml^−1^ EGF-Al647 (white) and after removing unbound ligand chased for the indicated amount of time. Immunofluorescence staining and colocalization analysis establishes the endocytic trafficking of EGF ligand through the degradative pathway from early (EEA1 and RAB5 compartments) to late (RAB7 and LAMP1 compartments) within 45 min. Green and red arrows: EGF-Al647 colocalizing with early or late markers, respectively. MCC: Overlap of EGF with endocytic markers. One-way ANOVA EEA1: *p* = 0.0056; RAB5: n.s; RAB7: n.s.; LAMP1:*p* = 0.0014; *n* = 3–4 confocal experiments with 3–4 images (each 4–5 cells) per condition. Shown are details. Data are mean ± SEM. **c** Pulse-chase experiment as in **b**, but analyzing ESCRT colocalization with EGF-Al647. ESCRT-0 (HRS), ESCRT-III (CHMP4B) and the Bro1 domain protein HD-PTP all show a maximum overlap with EGF at 15 min after stimulation, indicating a concerted recruitment to endosomes. Arrows indicate colocalization between EGF and ESCRTs. One-way ANOVA HRS: *p* < 0.0001; HD-PTP: *p* = 0.0072; CHMP4B: n.s.; *n* = 4–6 confocal experiments with 3–4 images (each 4–5 cells) per condition. Shown are details from those images. Data are mean ± SEM. **d** Pulse-chase experiment as in **b**, but analyzing the very early time points after EGF stimulation. HRS colocalization with EGF increases from 2 min onward, and CHMP4B is clearly detected 5 min after the EGF pulse. *t*-test, **p < 0.01; *n* = 3–4 experiments with 3–4 images (each 4–5 cells) per condition. Data are mean ± SEM. All scale bars, 5 µm

The ESCRT-0 component, hepatocyte growth factor receptor substrate (HRS), was reported to localize to early endocytic vesicles (SNX15-, RAB5- and EEA1-positive early endosomes)^26,27^. Since the ESCRT-III component CHMP4B was found both on early and late endocytic compartments^26,28,29^, we wondered about its localization in relation to ESCRT-0. We detected CHMP4B-GFP preferentially in early (EEA1- and HRS-positive) compartments, when compared to RAB7 and LAMP1 late endocytic compartments (Fig. 1a). Since ESCRTs are engaged in the sorting of activated epidermal growth factor receptors (EGFRs) into MVEs, we next investigated the localization of endogenous ESCRT components to endosomes after epidermal growth factor (EGF) stimulation. We established the flow of EGF ligand through the degradative pathway by pulse-chase experiments followed by analysis of co-occurrence with endosomal markers. After 5 and 15 min chase, the overlap of EGF was highest with the early endocytic markers EEA1 and RAB5. After 30 min, EGF reached late endocytic (RAB7-positive) compartments and after 45 min lysosomal (LAMP1-positive) compartments (Fig. 1b). The maximum overlap of EGF with the ESCRT proteins HRS, CHMP4B (charged multivesicular body protein 4b) and the Bro1 domain protein HD-PTP (histidine domain-containing protein tyrosine phosphatase) occurred 15 min after EGF stimulation (Fig. 1c), indicating that ESCRT-0–III and associated proteins localize mostly to RAB5- and EEA1-positive compartments and do not segregate between early compartments for “early” ESCRTs and late compartments for “late” ESCRTs. To elucidate how early after an EGF pulse ESCRTs can be detected at endosomes, we chased for 2, 3, 4 and 5 min and observed a gradual increase in EGF co-occurrence with HRS from as early as 2 min after EGF stimulation, while CHMP4B localization was clearly detectable from 5 min on (Fig. 1d). We conclude from these results that “early” and “late” ESCRTs as well as HD-PTP localize preferentially to early endocytic compartments.

### The main activity of ESCRT proteins is early after EGFR activation

To increase the temporal resolution of our analysis we decided to utilize fluorescently tagged ESCRT proteins for live-cell microscopy experiments. For this purpose we used the existing stable cell lines HeLa-CHMP4B-GFP and HeLa-CHMP3-GFP^30^ and generated additional stable HeLa cell lines with close to endogenous expression by using lentiviral vectors. Expression levels of ESCRT-expressing cell lines are shown in Supplementary Fig. 1 and within the following experiments. We also verified that the expression of tagged ESCRT proteins did not increase the number of bi- or multinucleate cells or affected their proliferation (Supplementary Figure 2). To additionally test the functionality of the tagged ESCRT proteins, we performed rescue experiments. Depletion of endogenous CHMP4B resulted in an inhibition of EGFR degradation, which could be rescued in the cell line stably expressing small interfering RNA (siRNA)-resistant CHMP4B-GFP (Supplementary Fig. 3A–D). Importantly, the presence of CHMP4B-GFP did not delay EGFR degradation in the presence of endogenous CHMP4B, despite a slightly higher expression than endogenous levels (Supplementary Fig. 3A–D). The same rescue setup also validated the functionality of our mCherry-HRS stably expressing cell lines (Supplementary Fig. 3E, F).

**Fig. 2:**
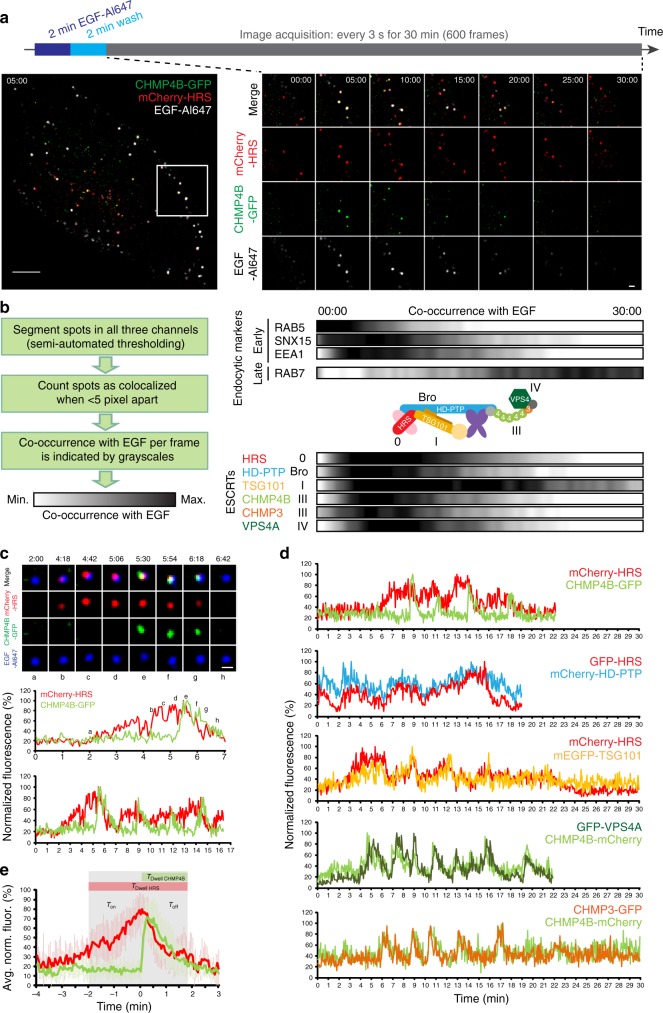
ESCRT proteins act concertedly 5 to 15 min after EGF stimulation. **a** Experimental setup and representative images from a movie showing the uptake of EGF-Al647 into endocytic vesicles. Scale bars, 5 µm (left) and 1 µm (right). **b** Quantification of endosomal localization was done by segmenting spots in all three fluorescence channels by semi-automated thresholding on complete frames which typically contain one or two HeLa cells. Spots below a threshold distance of <5 pixels (400 nm) were counted as co-occurring with EGF and this is visualized on a gray scale with white displaying minimal and black maximal co-occurrence. As expected, early endocytic markers showed maximal co-occurrence with EGF at early time points after starting the live-cell imaging, and late markers at later time points. Representatives of the ESCRT subcomplexes are color-coded according to the schematic. RAB5: 6 exp; SNX15: 5 exp.; EEA1: 7 exp.; RAB7: 6 exp; HRS: 55 exp.; HD-PTP: 7 exp.; TSG101: 7 exp.; CHMP4B: 32 exp.; CHMP3: 14 exp.; VPS4A: 13 exp. **c** Tracking of individual EGF-positive endosomes shows recruitment and dissociation of mCherry-HRS and CHMP4B-GFP and their normalized fluorescence intensity over time. Images of one representative endosome at indicated time points and corresponding fluorescence intensity measurement over time (in min). Representative example from >30 tracks from 9 independent experiments. Scale bar, 0.5 µm. **d** Tracking of individual EGF-positive endosomes in cell lines stably expressing combinations of ESCRT-0 (HRS) or ESCRT-III (CHMP4B) with other ESCRT proteins. Normalized fluorescence intensities over time of one representative track out of≥25 tracks from ≥4 independent experiments per combination. Tracks are displayed until the endosome went out of focus. Note the coordinated recruitment and dissociation of ESCRT proteins and that they show either slow and gradual waves (HRS, HD-PTP, TSG101) or rapid and transient kinetics (CHMP4B, CHMP3, VPS4A). **e** A total of 23 isolated fluorescence profiles of coordinated HRS and CHMP4B recruitment were individually normalized between 0 and 100 and then averaged. Mean intensity profiles of ESCRT waves ± SD. Data from 14 tracked endosomes, 6 independent live-cell imaging experiments

For live-cell imaging experiments we added fluorescently labeled EGF ligand for 2 min to HeLa cells stably expressing combinations of fluorescently tagged endocytic markers or ESCRT proteins. After washing away unbound ligand, images were acquired for 30 min with one frame taken every 3 s in all three channels (Fig. 2a, Supplementary Movie 1). In this way we could follow the EGF ligand through the degradative pathway as marked by early (RAB5, SNX15, EEA1) and late (RAB7) endocytic proteins^31,32^. To quantify EGF coinciding with endocytic markers or ESCRT proteins over time, we counted the average number of co-occurring spots per frame as outlined in Fig. 2b. As expected, we observed the highest overlap of EGF with early endocytic markers early after onset of imaging and with the late marker RAB7 later (Fig. 2b), in line with the results from the fixed-cell imaging (Fig. 1b).

We succeeded in visualizing tagged subunits from ESCRT-0 (HRS), ESCRT-I (TSG101), ESCRT-III (CHMP4B, CHMP3) as well as VPS4 and the Bro1 domain protein HD-PTP on endosomes and analyzed their co-occurrence with EGF over time. Strikingly, all of the analyzed ESCRTs and associated proteins had their maximal co-occurrence with EGF within the first 15 min of imaging (Fig. 2b). Only TSG101 showed a prolonged co-occurrence with EGF, which may reflect its internalization into ILVs^33^. These findings indicate that “early” and “late” ESCRT proteins have their main activity early after EGFR stimulation and that the different ESCRT subcomplexes may be able to coincide on the same endosomal compartment.

### ESCRTs show concerted and repetitive recruitment dynamics

To investigate the recruitment and disappearance of “early” and “late” ESCRTs on the same endosomal compartment in detail, we profited from the fluorescently labeled cargo, which we could follow up to 30 min in live-cell imaging experiments. We tracked individual EGF-positive vesicles from their endocytic uptake through the degradative pathway. We observed that mCherry-HRS (ESCRT-0) and CHMP4B-GFP (ESCRT-III) coincided transiently on the same endosome (Fig. 2c, upper panel). Fluorescence intensity measurements revealed a gradual accumulation and dissociation of mCherry-HRS, and an abrupt rise and gradual decline in CHMP4B-GFP fluorescence (Fig. 2c middle panel, Supplementary Movie 2). While both proteins dissociated synchronously, the recruitment dynamics followed distinct kinetics. Tracking the same vesicle for a longer period of time showed several coordinated fluorescence peaks, where CHMP4B was recruited each time when HRS had reached a maximum in its fluorescence intensity at the endosome (Fig. 2c lower panel).

Investigating other ESCRT components revealed that their kinetics either resembled those of HRS, showing slow and undulating waves (TSG101, HD-PTP) or the fast and transient CHMP4B kinetics (CHMP3 and VPS4A) (Fig. 2d). This divided the ESCRT subunits into two kinetically distinct groups, with ESCRT-0, -I and Bro1 domain proteins belonging to the “slow type” and ESCRT-III and VPS4A to the “transient” type of endosome localization dynamics. Typically the ESCRT activity on endosomes ceased before the end of the 30 min time lapse, which is also in line with the frame-by-frame analyses of our live-imaging data (Fig. 2b), placing the main ESCRT activity to the early time points after EGF stimulation.

To describe the coordinated ESCRT kinetics on endosomes, we averaged 23 isolated profiles from several tracked endosomes displaying HRS and CHMP4B (Fig. 2e). HRS showed an average dwell time (the time from recruitment onset until dissociation) of 195 ± 67 s (SD) and CHMP4B 80 ± 29 s (SD). The onset kinetics differed significantly, with HRS showing a slow and linear accumulation over 122 ± 50 s (SD) and CHMP4B a rapid accumulation over 12 ± 5 s (SD). The dissociation kinetics resembled each other with *t*_off_ (HRS) = 73 ± 25 s (SD) and *t*_off_ (CHMP4B) = 68 ± 27 s (SD) and occurred synchronously, indicating coordinated release of ESCRT-0 and ESCRT-III. Of note, whereas CHMP4B dissociated completely, HRS often did not reach baseline fluorescence despite showing a clear fluorescence decrease. Taken together, these data indicate that cargo sorting (ESCRT-0) and membrane remodeling (ESCRT-III) are temporally coordinated. In the following, we will term these characteristic coordinated fluorescence profiles “ESCRT waves”.

### One ESCRT wave results in the formation of a single ILV at a time

A key question is whether one ESCRT recruitment wave corresponds to the formation of one or several ILVs at a time. To investigate ILV formation at the ultrastructural level, we marked newly formed endosomes by labeling surface EGFR with an antibody recognizing the extracellular part of EGFR, followed by protein A conjugated with 10 nm gold (PAG10) labeling on ice (Supplementary Fig. 4A). The labeling neither impaired EGFR degradation nor led to EGF-independent EGFR degradation due to possible receptor crosslinking (Supplementary Fig. 4B). We stimulated endocytic uptake of EGFR by adding EGF ligand for defined periods of time before high-pressure freezing, freeze substitution and electron microscopy (EM).

**Fig. 3:**
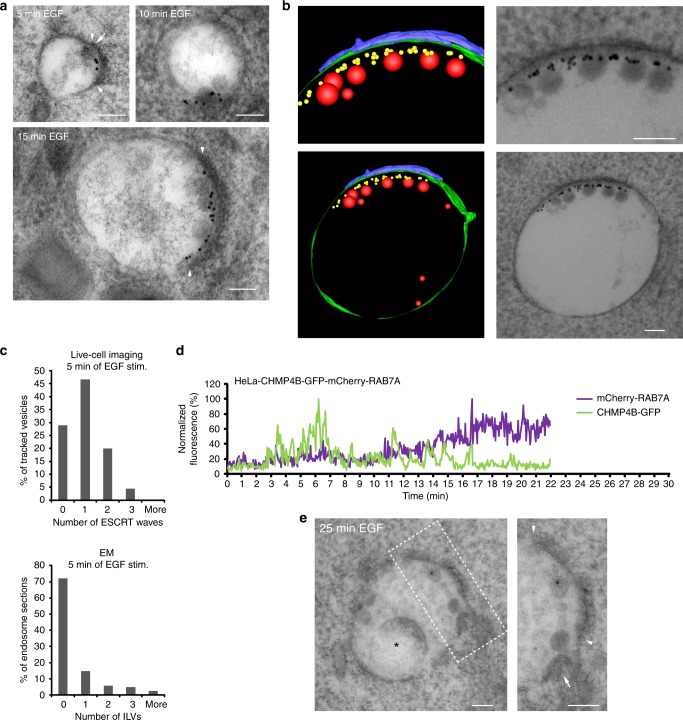
One ESCRT recruitment wave results in the formation of a single ILV at a time. **a** Representative electron micrographs of endosomes from HeLa cells stimulated for 5, 10 or 15 min with EGF. The 10 nm gold particles mark newly internalized EGFR (see also Supplementary Fig. 4A). Note an ILV budding profile in the 5 min sample (arrow) and an increasing number of ILVs formed over time, always in proximity to an endosomal EGFR/HRS/clathrin coat (boundaries marked by arrowheads). **b** Electron tomography of a 250 nm thick section of a gold-labeled endosome with several ILVs. Note that completely abscised ILVs (red) are found in close proximity to the endosomal EGFR/HRS/clathrin coat (blue). **c** Quantification of the number of ESCRT waves after 5 min of EGF stimulation (upper histogram) and quantification of the number of ILVs observed per endosome section in electron microscopy after 5 min of EGF stimulation (lower histogram). ESCRT waves were counted from 64 endosomes from 21 independent live-cell imaging experiments. For the EM analysis at least 100 gold-labeled endosomes were analyzed from 3 independent samples. See also Supplementary Fig. 4 F, G, H. **d** Fluorescence intensity profile of a tracked EGF-positive endosome over time from a CHMP4B-GFP and mCherry-RAB7 stably expressing HeLa cell. Note that CHMP4B-GFP flashing continues after the endosome has acquired RAB7. The track is one example from 15 tracks in total from 5 independent live-cell imaging experiments. **e** Electron micrograph of a gold-labeled endosome 25 min after EGF stimulation. Note that this endosome shows an ILV budding profile (arrow) and at the same time a degradative structure (asterisk). Arrowheads indicate the boundaries of an EGFR/HRS/clathrin coat. All scale bars, 100 nm

We decided to count ILVs between 40 and 60 nm diameter, since ESCRT-dependent ILVs in human cells were found to be >40 nm^34^ and the average size of ILVs was shown to be around 50 nm^35^, which was also in line with our measurements (Supplementary Fig. 4C). We observed newly formed (gold labeled) ILVs already 5 min after EGF stimulation, confirming the surprisingly early onset of ESCRT activity after endocytosis (Fig. 3a). At later time points, endosomes typically contained multiple ILVs. Of note, abscised ILVs accumulated predominantly in proximity to the EGFR-containing HRS/clathrin coat (Fig. 3a, b, Supplementary Fig. 4D), which marks the sorting microdomain of endosomes and which appears as an electron-dense structure at the limiting membrane of endosomes^5,36,37^. Importantly, we also observed ILVs directly under the limiting membrane in unlabeled endosomes, arguing against a simple tethering of ILVs by receptors via the anti-EGFR antibodies.

Next we counted the number of ESCRT waves in the first 5 min of live-cell imaging and correlated them to the number of ILVs in sections of gold-labeled endosomes observed in EM. From 64 analyzed endosome tracks, we observed on average 1.0 ± 0.8 waves in the first 5 min (Fig. 3c). We observed on average 0.5 ILVs per 150 nm endosome section, where 87% of the sections had zero or one ILV (Fig. 3c). The average diameter of a gold-labeled endosome after 5 min of EGF stimulation was 251 nm ± 129 (SD) (Supplementary Fig. 4E). Since an EM section of 150 nm thickness covers about half the volume of a 5 min endosome, the number of observed ILVs is lower than the number of ESCRT waves per endosome, as expected. Taken together, this speaks against a synchronized formation of several ILVs per ESCRT wave.

Extending this correlation for 10 and 15 min showed a gradual increase in the average numbers of ILVs per endosome section (Supplementary Fig. 3F, G), which indicates a linear increase in ILVs formed over time. From 20 min onwards some of the gold-labeled endosomes showed degradative structures precluding any further ILV quantification analysis. The number of ILVs observed by EM at 5, 10 and 15 min after EGF stimulation correlated well with the number of waves observed from live-cell imaging at the same time points (Supplementary Fig. 3F, H) and since we never observed more than one budding profile in association with the EGFR-containing HRS/clathrin coat, we conclude that one ESCRT recruitment wave reflects the formation of one single ILV at a time.

### ESCRT-dependent ILV formation is independent of endosome maturation

From our co-occurrence analysis we observed the main ESCRT activity from early on until approximately 15–20 min of live-cell imaging, while the RAB7 endosome maturation switch seemed to occur from approximately 15 min and onwards (Fig. 2b). To investigate whether ESCRT recruitment would be coordinated with endosome maturation, we tracked EGF-positive endosomes from a CHMP4B-GFP and mCherry-RAB7 double stable cell line. We frequently observed that CHMP4B recruitment continued on endosomes which had acquired RAB7 (Fig. 3d). In addition, we could also observe ILV budding profiles on the limiting membrane of endosomes, which contained degradative structures (Fig. 3e), confirming that ILV formation and endosome maturation/acidification can occur in parallel and most likely independently from each other.

### Clathrin recruitment to endosomes is required for normal ESCRT kinetics

**Fig. 4:**
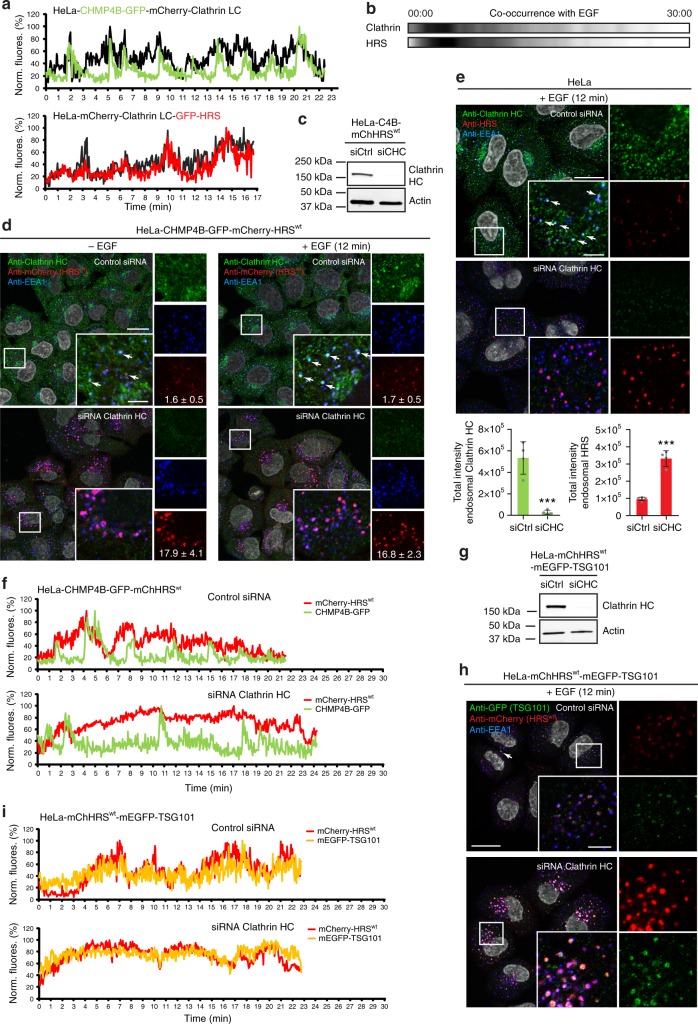
Depletion of clathrin leads to hyperstabilization of HRS and TSG101 on endosomes. **a** Fluorescence intensity profiles over time. Representative from 11 (HRS/Clathrin LC) and 13 (CHMP4B/Clathrin LC) tracks in total from 5 (HRS/Clathrin LC) and 4 (CHMP4B/Clathrin LC) independent live-cell imaging experiments. **b** Frame-by-frame colocalization analysis of clathrin and HRS with EGF over time. See also Fig. 2b for details. Data from 8 independent experiments. **c** Western blot (WB) showing KD of endogenous clathrin HC (CHC). **d** Immunostaining reveals a hyperrecruitment of mCherry-HRS to endosomes depleted for clathrin HC in cells stimulated or non-stimulated with EGF. Total fluorescence intensities of endosomal mCherry-HRS per cell quantified by high-content microscopy and indicated as AU (×10^5^) ± SD. Data from >2500 cells per condition from 4 independent experiments. Ctrl (−EGF) versus siCHC (−EGF): *p* < 0.001; Ctrl (+EGF) versus siCHC (+EGF): *p* < 0.001 (*t*-test). Arrows: mCherry-HRS and clathrin-positive EEA1 endosomes. **e** Immunostaining reveals a hyperrecruitment of endgenous HRS to endosomes depleted for clathrin HC. Quantification was done as in **d**. Data from >2500 cells per condition from 4 independent experiments. *t*-test: ***p < 0.001. Arrows: mCherry-HRS- and clathrin-positive EEA1 endosomes. **f** Live-cell imaging and fluorescence intensity profiles of tracked EGF-positive endosomes over time show a prolonged recruitment of mCherry-HRS to endosomes in clathrin HC-depleted cells. Representative examples of 12 (control) and 23 (siRNA clathrin HC) tracks from at least 4 independent experiments. **g** WB showing KD of endogenous clathrin HC (CHC). **h** Immunostaining reveals a hyperrecruitment of mEGFP-TSG101 to endosomes depleted for clathrin HC. Arrow: mEGFP-TSG101 localizing to a midbody. **i** Live-cell imaging and fluorescence intensity profiles of tracked EGF-positive endosomes over time show a prolonged recruitment of mEGFP-TSG101 and mCherry-HRS to endosomes in clathrin HC-depleted cells. Representative examples of 13 (control siRNA) and 15 (siRNA clathrin HC) tracks from at least 4 independent experiments. Scale bars, 20 µm and 5 µm (insets)

In mammalian cells clathrin is recruited to the same microdomains as HRS on endosomes^3,5,37^. To elucidate the kinetics of clathrin recruitment to endosomes, we utilized the same live-cell imaging setup and analysis as introduced in Fig. 2a. Clathrin dynamics were coordinated with CHMP4B dynamics and resembled HRS dynamics very closely (Fig. 4a, b) as expected since HRS recruits clathrin to endosomes^3,37^.

Next, we asked whether interference with clathrin recruitment to the sorting microdomain of endosomes would alter ESCRT dynamics. We therefore depleted clathrin by siRNA in cells expressing mCherry-HRS (Fig. 4c). To our surprise, mCherry-HRS was hyperrecruited to endosomes upon knockdown (KD) of clathrin and this was independent of EGF stimulation (Fig. 4d). Importantly, endogenous HRS also accumulated on endosomes in clathrin-depleted cells (Fig. 4e). Lack of clathrin led to an impaired uptake of EGF-Al647; however, increasing the EGF concentration resulted in modest uptake of fluorescent EGF allowing tracking of endosomes in mCherry-HRS- and CHMP4B-GFP-expressing cells. In line with the fixed-cell imaging (Fig. 4d, e), tracking of individual endosomes indicated a prolonged recruitment of HRS in every wave, while CHMP4B still showed transient dynamics (Fig. 4f). To investigate whether other ESCRT complexes would be affected by clathrin depletion, we performed KD experiments in mEGFP-TSG101-expressing cells (Fig. 4g). Fixed and live-cell imaging showed a stabilization of mEGFP-TSG101, indicating that TSG101 dynamics are similiarly affected by the absence of clathrin on endosomes as HRS kinetics (Fig. 4h, i).

**Fig. 5:**
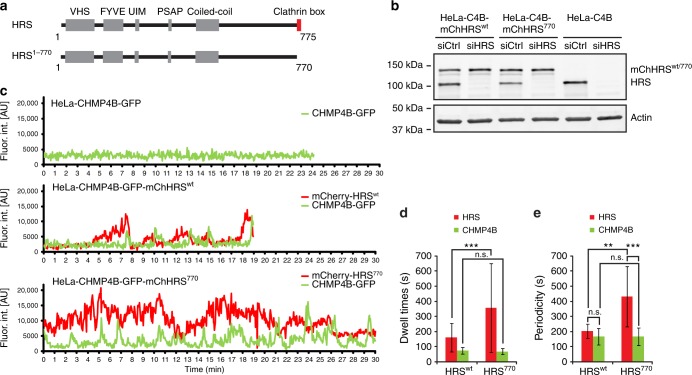
Absence of endosomal clathrin leads to hyperstabilization of HRS on endosomes. **a** Domain structure of HRS and the HRS mutant HRS^770^ lacking the clathrin-binding domain. **b** WB showing depletion of endogenous HRS and expression levels of siRNA-resistant mCherry-HRS^wt^ or mCherry-HRS^770^ in the background of the HeLa-CHMP4B-GFP cell line (“C4B”). **c** HeLa cells stably expressing CHMP4B-GFP alone or in combination with mCherry-HRS^wt^ or -HRS^770^ were depleted for endogenous HRS by siRNA as shown in Fig. 5b. Live-cell imaging and fluorescence intensity profiles of tracked EGF-positive endosomes over time. Representative examples of 13 (HRS^wt^), 12 (HRS^770^) and 8 (no HRS) tracks from ≥3 independent experiments. **d** Dwell times for individual mCherry-HRS or CHMP4B-GFP waves were quantified manually in a blinded way. HRS^wt^: 62 (HRS) and 55 (CHMP4B) waves, HRS^770^: 29 (HRS^770^) and 83 (CHMP4B) waves from the dataset described in **c**. Error bars indicate SD. *t*-test, ***p < 0.001, n.s. not significant. **e** Periodicities for mCherry-HRS or CHMP4B-GFP waves were quantified manually in a blinded way. Only tracks covering at least 200 frames were included. HRS^wt^: 11 tracks, HRS^770^: 11 tracks from the dataset described in **c**. Error bars indicate SD. *t*-test, **p < 0.01, ***p < 0.001, n.s. not statistically significant

To verify our findings from the clathrin KD experiments and to specifically abolish clathrin targeting to endosomes without disturbing clathrin functions elsewhere, we deleted the clathrin box of HRS (Fig. 5a) and generated stable cell lines expressing siRNA-resistant HRS^wt^ or HRS^770^. Efficient depletion of endogenous HRS resulted in replacement of the endogenous HRS for HRS^wt^ or HRS^770^ at close to endogenous levels (Fig. 5b), and the following experiments were done in cells depleted for endogenous HRS. The HRS^770^ deletion mutant was shown previously to be unable to bind clathrin^3,37^ and we verified this by immunofluorescence (IF) stainings followed by quantitative co-occurrence analysis of fixed cells, co-immunoprecipitation and live-cell imaging experiments (Supplementary Fig. 5A, B, C, D). In contrast to HRS^wt^, HRS^770^ was not able to recruit clathrin to endosomes as expected (Supplementary Fig. 5A, B, C, D^3,37^).

In the parental cell line, depletion of endogenous HRS resulted in CHMP4B not being recruited to endosomes as expected (Fig. 5c, upper panel). Expression of mCherry-HRS^wt^ rescued the recruitment of CHMP4B-GFP (Fig. 5c, middle panel), while mCherry-HRS^770^ was found strongly enriched on endosomes (Supplementary Fig 5E), mimicking the clathrin KD phenotype (Fig. 4d, e). Tracking of individual endosomes indicated a prolonged recruitment of HRS^770^ in every wave while CHMP4B kinetics appeared to be unaffected (Fig. 5c, lower panel), again mimicking the clathrin depletion (Fig. 4f). Indeed, quantification of dwell times (Fig. 5d) and periodicities (Fig. 5e) showed a longer persistence of HRS^770^ compared to HRS^wt^. Whereas similar periodicity measurements for HRS and CHMP4B in the HRS^wt^-expressing cells underline their coordinated recruitment pattern, there was a clear discrepancy in their periodicities in the HRS^770^ cells (Fig. 5e), which underlines the prolonged persistence of HRS^770^, but not CHMP4B, on endosomes. This indicates that clathrin regulates the dissociation of ESCRT-0 (HRS) and ESCRT-I (TSG101), but not ESCRT-III (CHMP4B).

### Clathrin recruitment to endosomes affects endosomal PtdIns3P levels

**Fig. 6:**
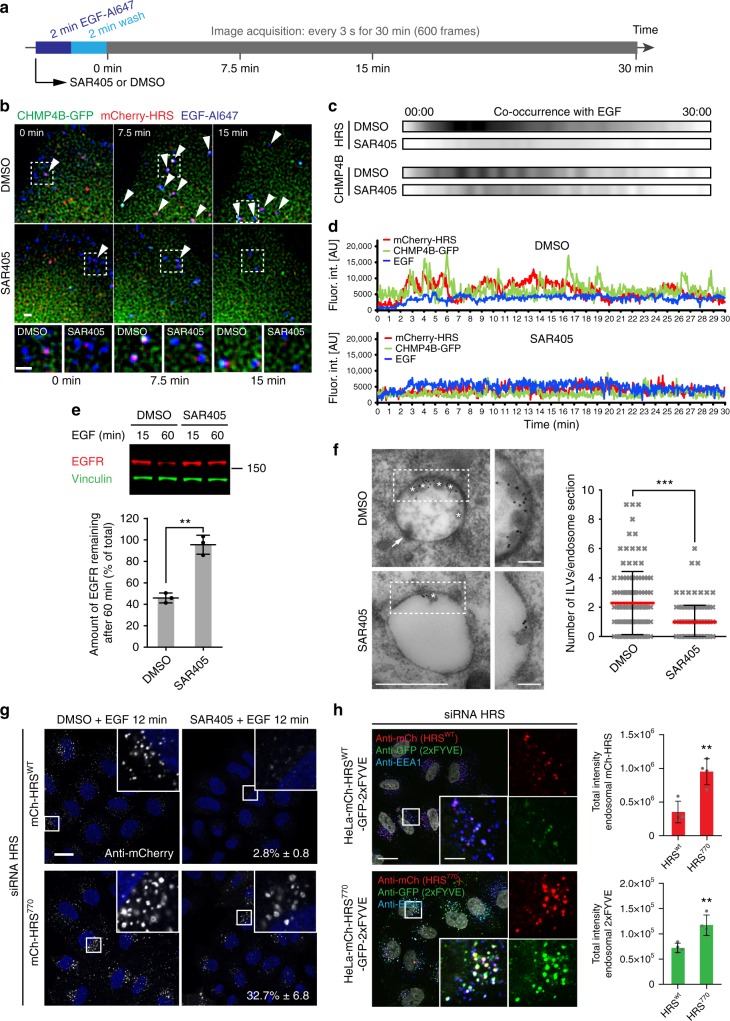
Lack of clathrin recruitment to endosomes increases PtdIns3P levels. **a** Experimental setup as in Fig. 2a, but with addition of DMSO or SAR405. **b** Representative images from live-cell imaging show that SAR405 treatment impairs HRS and CHMP4B localization to endosomes (arrowheads). Scale bars, 1 µm. **c** Frame-by-frame co-occurrence analysis (see also Fig. 2b) shows a drastically reduced overlap between HRS or CHMP4B with EGF upon SAR405 treatment. Data from 6 (DMSO) and 5 (SAR405) independent experiments. Co-occurrence normalized to the maximum of the DMSO control for HRS and CHMP4B. Note that CHMP4B shows a very transient localization to endosomes and thus only few spots can be observed at any time point, and therefore the reduction in co-occurrence upon SAR405 treatment appears not as clear as for HRS, which has a larger “dynamic range” due to its more stable association to endosomes. **d** Fluorescence intensity profiles over time show normal ESCRT waves in the presence of DMSO and lack of recruitment of HRS or CHMP4B in the presence of SAR405. **e** Quantitative WB analysis shows severely impaired EGFR degradation upon SAR405 treatment. Mean ± SD of three independent experiments. *t*-test, **p < 0.01. **f** SAR405 treatment leads to a drastic reduction in ILV formation. Electron micrographs depict representative endosomes 15 min after EGF stimulation. Asterisks: ILVs; arrow: forming ILV bud. Number of ILVs per endosome section from ≥100 gold-labeled endosomes per condition. Dot plot: mean ± SD. *t*-test, ***p < 0.001. Scale bar, 500 nm and 100 nm (inset). **g** Quantitative high-content microscopy shows an almost complete loss of mCherry-HRS^wt^ from endosomes upon SAR405 treatment. Also the majority of mCherry-HRS^770^ is lost upon SAR405 treatment, but a stable pool remains. Numbers: remaining intensity of endosomal HRS (% of control ± SD). Data from ≥1600 cells per condition from 4 individual image series. Scale bar, 20 µm. **h** Cells stably co-expressing mCherry-HRS^770^ show increased levels of GFP-2xFYVE on endosomes compared to mCherry-HRS^wt^. Total fluorescence intensities ± SD. Data from >4000 cells per condition from 4 independent experiments. *t*-test, **p < 0.01. Scale bar, 20 µm and 5 µm (inset)

ESCRT-0 recruitment to endosomes is dependent on phosphatidylinositol-3-phosphate (PtdIns3P), which binds the FYVE domain of HRS^27^. Therefore, we next tested whether the characteristic ESCRT waves observed by individual endosome tracking are PtdIns3P dependent. We acutely depleted PtdIns3P by inhibiting the PtdIns3P-kinase class III (VPS34) with the highly specific inhibitor SAR405^38^. As expected, HRS recruitment to EGF-positive endosomes was strongly reduced, as was the downstream ESCRT component CHMP4B (Fig. 6a–c). ESCRT waves were unchanged in the presence of dimethyl sulfoxide (DMSO), but absent in SAR405-treated cells (Fig. 6d) and EGFR degradation was severely impaired (Fig. 6e) in line with previous findings^39^.

To investigate ILV formation of SAR405-treated cells by electron microscopy, we stimulated endocytic uptake of EGFR by adding EGF ligand together with SAR405 or DMSO. In accordance with previously published results using the less specific phosphoinositide 3-kinase inhibitor Wortmannin^39–43^, endosome size increased slightly upon SAR405 treatment (Supplementary Fig. 6A) and SAR405-treated cells showed a strongly reduced number of ILVs after 15 min of EGF stimulation (Fig. 6f^39^). These findings verify a correlation between ESCRT waves and ILV formation and indicate that the repetitive ESCRT waves on endosomes reflect generation of MVEs.

Next, we asked whether the hyperstabilized HRS^770^ would be equally dependent on newly synthesized PtdIns3P as HRS^wt^. For this purpose, we incubated mCherry-HRS^wt^- and -HRS^770^-expressing cells depleted for endogenous HRS with DMSO or SAR405. HRS^wt^ was almost completely lost from endosomes upon SAR405 treatment as expected, whereas HRS^770^ was reduced to about one third of its intensity in the DMSO control as quantified by high-content microscopy (Fig. 6g). To elucidate which pool of HRS^770^ is SAR405 sensitive, we performed live-cell imaging experiments. Tracking newly formed endosomes with the help of EGF-Al647 uptake showed a lack of HRS^770^ recruitment to these vesicles in SAR405-treated cells (Supplementary Fig. 6B), similar to HRS^wt^ (Fig. 6d), while structures already positive for HRS^770^ showed a persistant HRS^770^ signal (Fig. 6g). We interpret these data as a requirement for PtdIns3P for the initial recruitment of ESCRT-0, while the hyperstabilized coat found in HRS^770^ seems to withstand the inhibition of the PtdIns3P-producing VPS34 enzyme. We therefore sought to investigate the levels of endosomal PtdIns3P by making use of the 2xFYVE probe, which binds specifically to PtdIns3P and reports the localization of this phospholipid^44^. We generated stable cell lines with low expression of GFP-2xFYVE (Supplementary Fig. 6C) in which PtdIns3P was found on early endosomes together with HRS^wt^ (Fig. 6h, Supplementary Fig. 6D) as reported before^45^. Interestingly, we observed severely increased levels of PtdIns3P in the HRS^770^ rescue situation (Fig. 6h) and also in clathrin-depleted cells (Supplementary Fig. 6D), consistent with the hyperstabilization of HRS in the absence of clathrin. These results point to a role of endosomal clathrin in regulating endosomal PtdIns3P turnover.

### Endosomal clathrin is required for efficient ILV formation

**Fig. 7:**
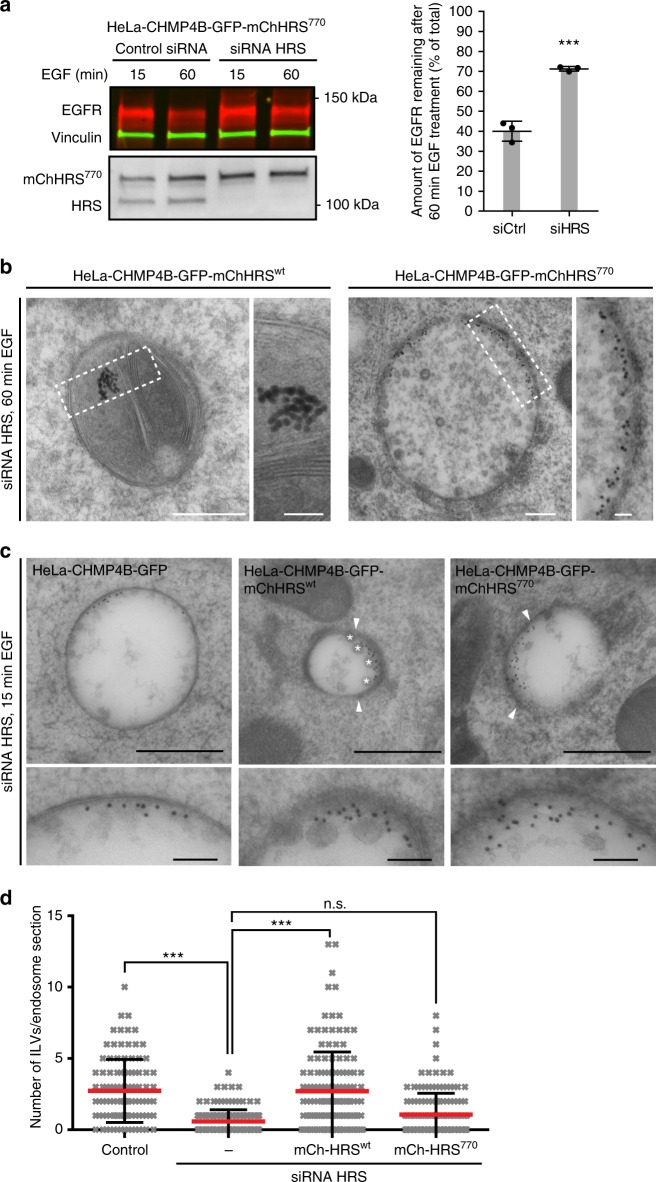
Endosomal clathrin is required for EGFR degradation, cargo sorting and efficient ILV formation. **a** mCherry-HRS^770^ does not rescue EGFR degradation upon depletion of endogenous HRS. Experiment was done side by side with Supplementary Fig. 3E,F. Mean ± SD from three experiments. *t*-test, ***p < 0.001. **b** Stably expressing HeLa cells as indicated were depleted for endogenous HRS as shown in Fig. 5b and processed for EM as described in Supplementary Fig. 4A. The 10 nm gold particles mark internalized EGFR after 60 min of EGF stimulation. Note that in HRS^wt^-expressing cells gold particles can be found as clusters inside degradative organelles, indicating degradation of gold-labeled EGFR. In contrast, in HRS^770^-expressing cells the gold-labeled EGFR is still found in the limiting membrane, where it covers a large portion of the surface, indicating impaired cargo sorting. Scale bar, 200 nm and 50 nm (inset). **c** Stably expressing HeLa cells as indicated were depleted for endogenous HRS as shown in Fig. 5b and processed for EM as described in Supplementary Fig. 4A. The 10 nm gold particles mark newly internalized EGFR after 15 min of EGF stimulation. Asterisks denote ILVs (40–60 nm diameter), arrowheads the boundaries of an endosomal EGFR/HRS/clathrin (HRS^wt^) or EGFR/HRS (HRS^770^) coat. Note the complete absence of this electron density in the absence of HRS (left panel). Scale bar, 500 nm and 100 nm (inset). **d** Quantification of the number of ILVs per endosome section. Data represented as dot plots with mean ± SD. Kruskal–Wallis test: *p* < 0.0001; Dunn’s multiple comparison test: ***p < 0.001, n.s. not statistically significant. More than 100 gold-labeled endosomes were analyzed per condition from at least 3 different samples per condition

To address whether the prolonged recruitment of HRS^770^ affects EGFR degradation, we performed rescue experiments. While the impaired EGFR degradation upon depletion of HRS could be completely rescued with HRS^wt^ (Supplementary Fig. 3E, F), the HRS^770^ mutant was not able to rescue the impaired EGFR degradation (Fig. 7a, Supplementary Fig. 7A) and showed an accumulation of EGFR in EEA1-positive compartments (Supplementary Fig. 7B)^37^. We next analyzed the localization of cargo by EM using the same experimental setup as described (Supplementary Fig. 4A). In control cells at 60 min after EGF stimulation the gold-labeled EGFR was found accumulated in the lumen of degradative organelles (Fig. 7b). In contrast, in HRS^770^-expressing cells, gold-labeled EGFR accumulated in the limiting membrane of endosomes (Fig. 7b). This could be due to impaired cargo sorting and/or ILV formation. We therefore performed EM experiments with 15 min of EGF stimulation to count the number of ILVs per 150 nm section of gold-labeled endosomes. Depletion of HRS led to a small increase in endosome size, as reported before^34^, and the same tendency was seen in the HRS^770^ mutant (Supplementary Fig. 7C). Importantly, the drastic reduction in the number of ILVs observed in HRS-depleted cells^34^ could not be rescued by the HRS^770^ mutant (Fig. 7c, d). In contrast, HRS^wt^ showed a full rescue when compared to unperturbed cells (Fig. 7c, d). Of note, depletion of HRS led to an increased number of 20-40 nm small ILVs (Supplementary Fig. 7D), which likely represents upregulation of ESCRT-independent ILV formation as previously described^34,46^. Also, the HRS^770^ mutant showed a moderate increase in small ILVs (Supplementary Fig. 7D). These results indicate a role for clathrin in the formation of ESCRT-dependent ILVs, which is important for EGFR degradation.

### Clathrin governs inverse membrane remodeling on endosomes

**Fig. 8:**
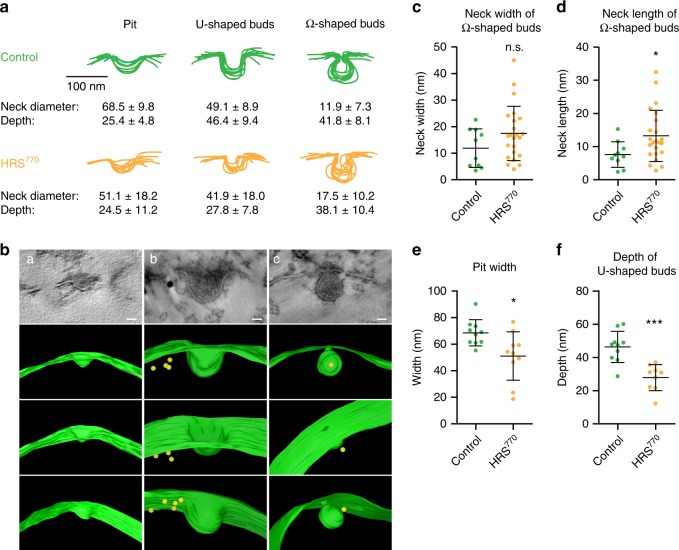
The absence of clathrin at endosomes results in aberrant ILV formation. **a** Budding profiles as observed in electron micrographs were binned into three morphological categories: Pits, U-shaped buds and omega-shaped buds (see also Supplementary Fig. 8). The outlines of these budding profiles were aligned with each other to assess their uniformity. Upper row: budding profiles from the control dataset. Lower row: budding profiles from the siHRS/HRS^770^ dataset. In all, 9–10 profiles per category and condition were used for superimposition. Neck diameter and depth were measured on all displayed profiles and their average ± SD is indicated. **b** Tomograms showing examples of every budding profile category. Scale bar, 20 nm. **c** Neck width of Omega-shaped buds represented as dot plot. Mean ± SD. *t*-test, n.s. not statistically significant. **d** Neck length of Omega-shaped buds represented as dot plot. Mean ± SD. *t*-test, *p < 0.05. **e** Pit width represented as dot plot. Mean ± SD. *t*-test, *p < 0.05. **f** Depth of U-shaped budding profiles represented as dot plot. Mean ± SD. *t-*test, ***p < 0.001

To understand the reason for the reduced number of ILVs in clathrin recruitment-deficient cells, we investigated newly forming ILV buds from 15 min of EGF-stimulated EM samples. In our control dataset we observed budding profiles in 7.4% of 343 sections of gold-labeled endosomes, while the HRS^770^ mutant dataset had 11.4% budding profiles from 368 endosome sections. Since we had seen a reduction in the number of formed ILVs (Fig. 7d), this increased number in budding ILVs may indicate a slowed process of ILV formation. Next we grouped the budding profiles according to their stage of ILV formation into three categories: shallow pits, U-shaped and omega-shaped profiles (Supplementary Fig. 8A). The budding profiles from the control showed surprisingly uniform shapes for each of these categories (Fig. 8a, b) illustrating that the membrane invaginates first as a broad and flat pit and then transforms into a U-shaped deep invagination. Of note, the depths of the U-shaped profiles (Fig. 8a) were similar to the final diameter of ILVs, which indicates that the membrane deforms first in depth and then constricts at the neck. Interestingly, we observed in many instances electron-dense material inside the forming ILV, which could reflect the presence of clathrin or ESCRT subunits (Fig. 8b, Supplementary Fig. 8), in line with published results^33,47^.

While we never observed more than one budding profile per microdomain in the control dataset, we found seven examples of two profiles in the same endosome section in the HRS^770^ dataset (Supplementary Fig. 8B). In particular, we observed almost three times more omega-shaped budding profiles in HRS^770^ compared to the control dataset (6.3% in HRS^770^ and 2.3% in control). In addition, they showed a tendency for a wider neck diameter (Fig. 8c) and a significantly longer neck (Fig. 8d) with often irregular morphology (Fig. 8a, Supplementary Fig. 8B). The existence of more and aberrant omega-shaped budding profiles in the HRS^770^ mutant, and the reduction in the number of ILVs, indicates that membrane constriction and ILV scission are perturbed.

Measuring the width of the pits showed a significantly reduced size of these shallow membrane invaginations in the HRS^770^ mutant (Fig. 8e). Importantly, the depth of the U-shaped budding profiles was also significantly reduced (Fig. 8f), which could implicate clathrin in defining the size of the future ILV. In addition, when counting the number of budding profiles with EGFR gold in vicinity to a forming ILV (closer than 40 nm), HRS^wt^ cells showed gold particles close to the bud in 43.3% of the cases, compared to only 16.6% in HRS^770^-expressing cells. This may indicate a failure to concentrate cargo in the absence of clathrin.

Taken together, clathrin governs the whole process of inverse membrane remodeling on the endosome from early invagination and cargo concentration to deeper membrane deformation and finally scission of ILVs.

## Discussion

The timing of endosomal ESCRT recruitment and ILV biogenesis has remained unknown, as has the mechanistic function of the endosomal clathrin coat. By following endosomes containing EGFR as a cargo, we have been able to combine live and electron microscopy to reveal that the formation of an MVE is mediated by characteristic and repetitive concerted recruitment waves of the whole ESCRT machinery at endosomes, starting immediately after cargo internalization. The highly coordinated recruitment of ESCRT-0, ESCRT-I, HD-PTP, ESCRT-III and VPS4 can ensure that the cargo, which is sequestered by ESCRT-0, will be efficiently sorted into forming ILVs. Since we find that one characteristic ESCRT wave corresponds to the formation of one ILV, we are now able to understand the timing of this process, and we find a surprising role for clathrin in governing ILV formation.

**Fig. 9:**
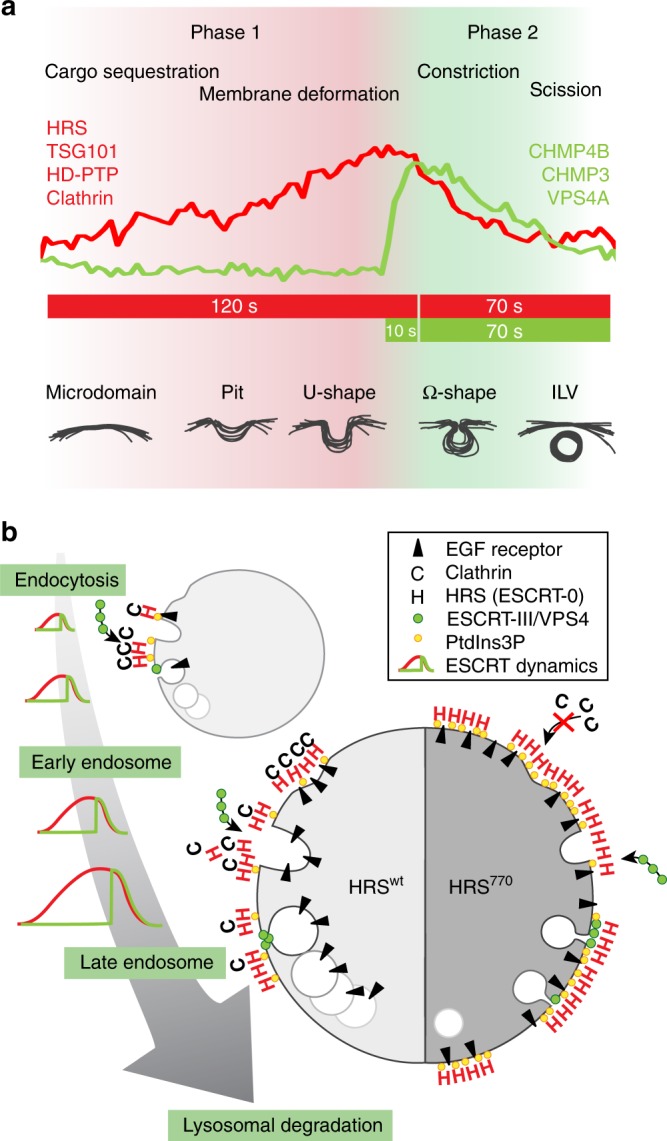
Models of ESCRT-dependent ILV formation. **a** Model combining ESCRT kinetics with the timing of ILV formation. Phase 1: HRS and clathrin slowly accumulate on the endosome membrane, where they sequester cargo into a sorting microdomain, visible as an electron-dense EGFR-containing HRS/clathrin coat. TSG101 and HD-PTP show similar undulating kinetics. Based on our findings with the clathrin-binding mutant of HRS, we suggest that the first phase comprises both cargo sorting and membrane deformation. Phase 2: ESCRT-III and VPS4A show a rapid accumulation which may reflect polymerization of ESCRT-III subunits and simultaneous recruitment of VPS4A, followed by a concerted dissociation of all ESCRT complexes. We suggest that the second phase may correspond to constriction and scission of ILVs. For details see Discussion. **b** Model of ESCRT-dependent ILV formation and the role of clathrin. Left: to ensure efficient cargo degradation in lysosomes, ESCRT proteins and clathrin are recruited to endosomes as they mature, in a coordinated and repetitive wave-like pattern. Each wave (which lasts for about 200 s) correlates with the formation of one ILV. Right: in the absence of endosomal clathrin, the wave dynamics are disturbed, ESCRT-0 and cargo accumulate on the endosomal membrane, and ILV formation is perturbed; the size of the forming bud is smaller, the forming ILV has a longer neck, and the diameter of the few ILVs that do form is smaller

To reconcile ESCRT kinetics with ILV formation, we suggest the following model (Fig. 9a).

Phase 1: The slow and linear accumulation of HRS on the endosomal membrane (duration approximately 120 s) reflects cargo sorting: ESCRT-0, together with clathrin, sequesters cargo on the limiting membrane of an endosome, forming a sorting microdomain which is visible as an electron-dense coat^5,36^ (Figs. 3a, b and 6f, Supplementary Fig. 4D). The ESCRT-I subunit TSG101 shows a similar slow and linear accumulation. We were not able to visualize ESCRT-II, but ESCRT-I was shown to form a supercomplex with ESCRT-II^6^, suggesting that ESCRT-II has a similarly slow recruitment. Since ESCRT-I/II were shown to deform membranes in vitro^48^, and ESCRT-0 and ESCRT-I are synchronously recruited to endosomes, we suggest that cargo sorting and membrane deformation may occur in parallel, starting in phase 1. This is strengthened by our finding that clathrin, which is also recruited in phase 1, is required for the normal size of pits and U-shaped buds. The endosomal clathrin coat is important for cargo concentration in microdomains^37^, and tightly bound to the membrane- and cargo-anchored HRS. It is tempting to speculate that clathrin might have a biomechanical role in cargo crowding leading to membrane deformation^49^. This could contribute to defining the size of the forming ILVs, which are smaller in the absence of clathrin. Therefore, this first phase could comprise both cargo sorting and membrane deformation, corresponding to appearance of visible coats, shallow pits or deeper U-shaped budding profiles, and typically lasting for approximately 120 s.

Phase 2: ESCRT-III (CHMP4B, CHMP3) and VPS4A show a rapid accumulation (duration typically 9–15 s). The initiation of recruitment could be promoted by a suitable negative membrane curvature^50^ generated by ESCRT-I/II, cargo crowding and clathrin in the first phase. The exponential increase probably reflects polymerization of ESCRT-III subunits and simultaneous multivalent recruitment of VPS4A. VPS4A drives constriction and scission of the bud necks^8^ and promotes the observed dissociation of ESCRT-III subunits (duration typically around 70 s). Importantly, ESCRT-0 dissociates simultaneously, and the concerted release of ESCRT-0, -I and ESCRT-III is required for efficient ILV formation, as demonstrated by the impaired ILV formation in the hyperstable HRS^770^ mutant. Therefore, this second phase most likely corresponds to the formation of omega-shaped budding profiles and ultimately scission of a nascent cargo containing ILV, and typically lasts for approximately 80 s.

Endosomes contain a non-canonical clathrin coat which was implicated in cargo sorting, but its molecular function has remained enigmatic^4,5,37^. Our finding that clathrin facilitates the timely dissociation of ESCRT-0 and ESCRT-I, and that this is important for efficient ILV formation was unexpected and opens a possibility to understanding the underlying mechanism of ESCRT-dependent ILV formation. Surprisingly, although HRS and TSG101 are stabilized on the endosome membrane in the absence of clathrin, ESCRT-III is still recruited normally. The recruitment of ESCRT-III, accumulation of aberrant omega-shaped budding profiles and reduction in the number of ILVs in the absence of clathrin indicates that membrane constriction and scission can occur, although less efficiently, perhaps impaired by steric hindrance from a hyperstable ESCRT-0/-I coat.

We observed that ILVs form and accumulate under the electron-dense EGFR-containing HRS/clathrin coat (Figs. 3a, b and 6f, Supplementary Fig. 4D). Whereas the coat is clearly defined and visible on HRS^wt^ endosomes, it was missing in HRS-depleted endosomes (Figs. 6f and 7c^34^). Importantly, HRS^770^ endosomes still displayed an electron-dense coat containing gold-labeled EGFR, in the absence of endosomal clathrin (Fig. 7c, Supplementary Fig. 8B). This indicates that the electron density is not defined by clathrin alone but also results from accumulation of ESCRT proteins and cargo. Interestingly, in omega-shaped budding profiles from control endosomes, we sometimes observed a local reduction in electron density above the constricted neck, although the coat was still present on both sides. In contrast, the coat was still prominent over the aberrant HRS^770^ omega profiles (Supplementary Fig. 8A, B), which could represent accumulated HRS and downstream ESCRTs in the absence of clathrin (Figs. 4, 5 and 6h). Our findings indicate that for efficient ILV formation, HRS needs to dissociate transiently at the site of ILV formation, presumably to release steric hindrance and allow efficient membrane constriction and scission by ESCRT-III and VPS4.

How could clathrin regulate the dissociation kinetics of HRS? One possibility would be that since HRS binds to ubiquitinated cargo^36^, the sorting defect of cargo in the absence of clathrin (Fig. 7b) may lead to a stable association of HRS on the endosome membrane. Additionally, tyrosine phosphorylation of HRS has been shown to facilitate its dissociation from endosomes^51^, and it is possible that clathrin indirectly regulates this process. Alternatively, in clathrin-mediated endocytosis, clathrin recruits factors that facilitate its uncoating from conventional clathrin-coated endosomes^52–56^ and this may happen in an analogous manner on the endosome membrane. HRS is recruited to endosomes by PtdIns3P^27^, which was clearly evident from the loss of ESCRT waves in SAR405-treated cells (Fig. 6d). We observed a stabilization of the PtdIns3P probe 2xFYVE in cells lacking clathrin recruitment to endosomes and with clathrin knockdown (Fig. 6h, Supplementary Fig. 6D), indicating a disturbed turnover of PtdIns3P in the absence of clathrin. It is therefore tempting to speculate that clathrin may recruit a protein that could lead to a transient and localized PtdIns3P turnover, either a PtdIns3P-phosphatase or -kinase. Indeed, clathrin and HRS follow the same dynamics (Fig. 4a, b), indicating that in a normal setting, they dissociate synchronously from endosomes.

Small ILVs have been previously described to derive from a CD63-dependent, ESCRT-independent mechanism^34,46^. When ESCRT localization to endosomes was abolished by acute depletion of PtdIns3P by SAR405, we did not observe the drastic increase in small <40 nm ILVs seen in cells depleted of HRS by siRNA. This could indicate a slow upregulation of ESCRT-independent ILV formation over time, which occurs in cells persistently devoid of ESCRT-dependent ILV formation, as suggested before^34^. These tiny ILVs were also not prominent in endosomes from control cells, indicating that ESCRT-dependent ILV formation is the favored process, or that the presence of the ESCRT coat inhibits the alternative pathway as suggested before^34^. In HRS-depleted cells expressing HRS^770^, where the endosomes had an electron-dense coat, we observed a small increase in the amount of small ILVs. If the presence of a coat is inhibitory for ESCRT-independent ILV formation, the small ILVs that form in the HRS^770^ mutant could still be ESCRT dependent. This is supported by our finding that the HRS^770^ mutant endosomes had smaller pits, shallow U-budding profiles and still recruited ESCRT-III, indicating that small ILVs can form in the absence of clathrin, although the overall ILV formation is perturbed in these cells. Clathrin has not been implicated during ILV formation in yeast^57^. Interestingly, yeast has smaller-sized ILVs (25 nm diameter)^8,58^ and it is tempting to speculate whether the absence of clathrin may be among the reasons for this size difference between mammalian and yeast ILVs.

In models of endosomal ESCRT function the actions of “early” and “late” ESCRTs have been suggested to occur sequentially and to involve a handover of cargo^1,2,6,59^. From localization studies, “early” ESCRTs like HRS have primarily been found at early endocytic structures^23,25–27^ and “late” ESCRTs like CHMP4B and CHMP3 have been found both together with early and late endocytic markers^26,28,29^. Some of these studies were done by transiently overexpressing tagged ESCRT proteins at relatively high level, which might affect their localization^23,25^. For this reason we decided to work with stable cell lines expressing close to endogenous levels (Supplementary Fig. 1) and to verify that the fluorescently tagged ESCRT proteins are functional by EGFR degradation rescue experiments (Supplementary Fig. 3). We found that “early” (ESCRT-0) and “late” (ESCRT-III) ESCRTs can both localize to early endocytic compartments (Figs. 1 and 2a, b). Our data clearly show that ESCRTs transiently localize to endosomes and can be detected there simultaneously and repeatedly, in parallel to endosome maturation starting immediately after EGF stimulation.

In yeast the ESCRT-III subunits and Vps4 reside on endosomes for about 3-45 s^60^. These rapid kinetics resemble the ones observed in our study for the rapid and transient recruitment of CHMP3, CHMP4B and VPS4A (dwell times approximately 80 s). In addition, we observed slow and linear recruitment dynamics for HRS, HD-PTP, TSG101 and clathrin. Interestingly, both the fast and the slow kinetics observed in this study resemble the ones observed during virus budding: while TSG101 and the Bro1 domain protein ALIX show a slow and linear recruitment to the recruiting viral Gag protein over up to 10 min, CHMP4B, CHMP4C, CHMP1B and VPS4 are recruited very rapidly and transiently (1–5 min) to Gag^12–15^. The coordinated recruitment of the viral Gag protein with ESCRTs results in the formation of one virion^61^. This stoichiometry and the kinetics are in line with our findings in MVE formation, but the initial recruitment of Gag together with TSG101 (or ALIX) seems to require slightly more time (10 min versus 2 min in ILV formation), which could reflect the larger dimensions during virus budding (HIV diameter ~120–150 nm^61,62^ versus ILV diameter ~50 nm Supplementary Fig. 4C^35^). Likewise, during cytokinetic abscission, an even larger constriction starting from about 1 µm is to be handled by the ESCRT machinery, which could correspond to the longer ESCRT recruitment timescale of approximately 1 h^16^.

In conclusion, we have established the dynamics and timing of ESCRT recruitment and ILV biogenesis (Fig. 9a) and uncovered novel functions for clathrin in these processes (Fig. 9b). Our results demonstrate how “early” and “late” ESCRTs cooperate to mediate endosomal cargo sorting and ILV budding and reveal important similarities and differences with other ESCRT-dependent processes. Although the involvement of clathrin and ESCRT-0 is unique to the endosomal sorting functions of ESCRTs, the recruiting functions of ESCRT-0 are paralleled by Gag in HIV budding and CEP55 in cytokinesis. It will be interesting to learn whether other ESCRT activities depend on clathrin-like scaffolds that control ESCRT dynamics and membrane remodeling.

## Methods

### Cell culture and generation of stable cell lines

HeLa (Kyoto) cells (obtained from D. Gerlich, Institute of Molecular Biotechnology, Wien, Austria) were grown according to ATCC guidelines in Dulbecco's modified Eagle's medium (DMEM) high glucose (Sigma-Aldrich) supplemented with 10% fetal calf serum, 100 U ml^−1^ penicillin, 100 µg ml^−1^ streptomycin and maintained at 37 °C under 5% CO_2_. Cell lines are authenticated by genotyping and regularly tested for mycoplasma contamination. Stable HeLa (Kyoto) cell lines expressing CHMP4B-GFP or CHMP3-GFP were obtained from A. Hyman (Max Planck Institute for Molecular Cell Biology and Genetics, Dresden, Germany^30^). All other stable cell lines were lentivirus-generated pools based on HeLa or HeLa-CHMP4B-GFP or HeLa-CHMP3-GFP, generated as described in ref. ^63^. The EGFP- and mCherry fusions were generated as Gateway pENTR-GFP and pENTR-mCherry plasmids by conventional restriction-enzyme-based cloning. From these vectors, lentiviral transfer vectors were generated by recombination into pLenti Destination vectors (Addgene plasmid number 17451, and vectors derived from pCDH-PGK-MCS-IRES-PURO or -BLAST (System Biosciences)) using Gateway LR reactions (Invitrogen). VSV-G pseudotyped lentiviral particles were packaged using a third-generation packaging system (Addgene plasmid numbers 12251, 12253 and 12259)^64^. Cells were then transduced with virus and stable expressing populations were generated by antibiotic selection. Some of the stable cell lines were sorted by flow cytometry to obtain pools of cells with suitable levels of expression. Detailed cloning procedures can be requested from the authors. Importantly, none of the cell lines showed any aberrations in proliferation or multinucleation as analyzed by flow cytometry (Supplementary Fig. 2). We used the following stable cell lines: HeLa-CHMP4B-GFP^30^, HeLa-CHMP4B-GFP-mCherry-HRS^wt^, HeLa-CHMP4B-GFP-mCherry-HRS^770^, HeLa-CHMP4B-GFP-mCherry-ClathrinLC, HeLa-CHMP4B-GFP-mCherry-RAB7, HeLa-CHMP3-GFP^30^, HeLa-CHMP3-GFP-CHMP4B-mCherry, HeLa-GFP-HRS-mCherry-HD-PTP, HeLa-GFP-HRS-mCherry-RAB5, HeLa-mCherry-HRS-GFP-SNX15, HeLa-mCherry-HRS-mEGFP-TSG101, HeLa-mCherry-HRS^wt^-GFP-2xFYVE, HeLa-mCherry-HRS^770^-GFP-2xFYVE, HeLa-GFP-VPS4A-CHMP4B-mCherry, HeLa-mCherry-ClathrinLC-GFP-HRS^wt^ and HeLa-mCherry-ClathrinLC-GFP-HRS^770^.

### Immunostaining, antibodies and reagents

Cells grown on coverslips were permeabilized with 0.05% saponin in PEM buffer (80 mM K-Pipes, pH 6.8, 5 mM EGTA, and 1 mM MgCl_2_) for 5–10 min on ice to decrease the fluorescent signal from the cytosolic pool of proteins before fixation in 3% formaldehyde for 15 min^65^. Cells were washed twice in phosphate-buffered saline (PBS) and once in PBS containing 0.05% saponin before staining with the indicated primary antibodies for 1 h. After washing three times in 0.05% saponin in PBS, cells were stained with secondary antibodies for 1 h, and washed three times in PBS. The cells were mounted in Mowiol containing 2 mg ml^−1^ Hoechst 33342 (Sigma-Aldrich).

Antibodies: mouse anti-GFP (clones 7.1 and 13.1, 11814460001, immunofluorescence 1:400, western blot 1:500), mouse anti-β-actin (A5316, western blot 1:10,000) and mouse anti-Vinculin (V9131, western blot 1:400) were from Sigma-Aldrich, human anti-EEA1 serum^66^, immunofluorescence 1:160,000) was a gift from Ban-Hock Toh, Melbourne, Australia, rabbit anti-HRS (immunofluorescence 1:100, western blot 1:1000) has been described previously^3^, mouse-anti-RAB5 (4F11, immunofluorescence 1:2500) was a gift from C. Bucci, University of Lecce, Italy, rabbit anti-RAB7 (D95F2, immunofluorescence 1:200) was from Cell Signaling Technology (9367), mouse anti-LAMP1 (H4A3, immunofluorescence 1:2500) was from the Developmental Studies Hybridoma Bank, rabbit anti-HD-PTP was from Proteintech (10472-1-AP, immunofluorescence 1:100), rabbit anti-CHMP4B was generated as described previously^67^ (immunofluorescence 1:500, western blot 1:1000), sheep anti-EGFR (20-ES04, immunofluorescence 1:4000, western blot 1:7000) was from Fitzgerald, mouse anti-EGFR (555996, extracellular labeling of EGFR) was from Pharmingen, goat-anti-mCherry (AB0040-200, immunofluorescence 1:400, western blot 1:1000) and mouse anti-clathrin HC (X-22, immunofluorescence 1:500) were from Acris Antibodies, rabbit anti-clathrin HC (ab21679, western blot 1:1000) was from Abcam. All secondary antibodies used for immunofluorescence studies were obtained from Jacksons ImmunoResearch Laboratories or from Molecular Probes (Life Technologies). Secondary antibodies used for western blotting were obtained from LI-COR Biosciences GmbH, and horseradish peroxidase-conjugated secondary antibodies were from Jackson. The working concentration was for SAR405 (A8883; ApexBio) 6 μM; for DMSO (D2650; Sigma-Aldrich) 0.2%.

### siRNA transfections

All siRNAs were purchased from Ambion® (Thermo Fisher Scientific) and contained the Silencer Select modification. Cells at 50% confluency were transfected using Lipofectamine RNAiMAX transfection reagent (Life Technologies) following the manufacturer’s instructions. Cells were transfected with 50 nM siRNA targeting human HRS (5′-GCACGUCUUUCCAGAAUUC-3′), human CHMP4B (5′-AGAAAGAAGAGGAGGACG-3′) or human clathrin HC (5′-AUCCAAUUCGAAGACCAAU-3′) for 5 days. The HRS and CHMP4B transgenes are mouse sequences and resistant towards these siRNAs. Non-targeting control Silencer Select siRNA (predesigned, catalog number 4390844) was used as control.

### Immunoblotting

Cells were washed with ice-cold PBS and lysed with 25 mM Hepes, pH 7.2 (H4034; Sigma-Aldrich), 125 mM potassium acetate (104820; Merck Millipore), 2.5 mM magnesium acetate (105819; Merck Millipore), 5 mM EGTA (E3889; Sigma-Aldrich), 0.5% Triton-X-100 (Sigma-Aldrich) and 1 mM dithiothreitol (DTT; D0632; Sigma-Aldrich) supplemented with protease inhibitor cocktail (P9340; Sigma-Aldrich) or lysed in 2× sample buffer (125 mM Tris-HCl, pH 6.8, 4% SDS, 20% glycerol, 200 mM DTT and 0.004% bromophenol blue). Lysates were subjected to sodium dodecyl sulfate–polyacrylamide gel electrophoresis on 10% or 4–20% gradient gels (mini-PROTEAN TGX; Bio-Rad). Proteins were transferred to polyvinylidene difluoride (PVDF) membranes (TransBlot® Turbo^TM^ LF PVDF, Bio-Rad) followed by antibody incubation in 2% bovine serum albumin in Tris-buffered saline with 0.1% Tween-20. Membranes incubated with fluorescent secondary antibodies (IRDye680 or IRDye800; LI-COR) were developed with an Odyssey infrared scanner (LI-COR), whereas those incubated with horseradish peroxidase-conjugated antibodies were developed using Clarity Western ECL substrate solutions (Bio-Rad) with a ChemiDoc XRS+ imaging system (Bio-Rad). Quantification of immunoblots was done using the Odyssey Software. Please see Supplementary Figs. 9–11 for uncropped membranes.

### Co-immunoprecipitations

HeLa cells stably expressing mCherry-HRS^wt^ or -HRS^770^ (HeLa-CHMP4B-GFP-mCherry-HRS^wt/770^) were stimulated for 12 min with EGF (50 ng ml^−1^) or not stimulated, washed twice in ice-cold PBS before lysis in 25 mM HEPES (pH 7.2), 125 mM potassium acetate, 2.5 mM magnesium acetate, 5 mM EGTA, 0.05% NP40, 1 mM DTT, protease inhibitor cocktail (Sigma-Aldrich), Phospho STOP (Sigma-Aldrich) and 500 mM NEM (Sigma-Aldrich). Lysates were centrifuged for 10 min at 16,000 × *g* and supernatants were immunoprecipitated with goat-anti-mCherry antibody (3 µg antibody per sample, Acris antibodies) and DynabeadsTM Protein G (10004D, Thermo Fisher) rotating for 20 min at 4 °C. The immunoprecipitates were washed three times in lysis buffer, eluted with 2× sample buffer and subjected to immunoblotting as described above.

### Pulse-chase experiments and colocalization analysis

For pulse-chase experiments, cells were stimulated for 2 min with 50 ng ml^−1^ EGF-Al647 (E35351, Thermo Fisher Scientific) and then washed with warm DMEM. After indicated chase times, cells were fixed and immunostained as described in “Immunostaining, antibodies and reagents”. Images were acquired by confocal fluorescence microscopy with 0.7 µm confocal sections at fixed intensity settings below saturation. Colocalization with EGF was quantified with the ImageJ plugin “JACoP”^68^ and Manders’ colocalization coefficient (MCC)^69^ was used to describe overlap of EGF with endocytic markers or ESCRT proteins. The same type of image acquisition and analysis was done for cells stimulated with 50 ng ml^−1^ EGF for 12 min to assess colocalization between HRS and clathrin.

### Confocal fluorescence microscopy

Confocal fluorescence microscopy was done with a Zeiss LSM 710 or 780 microscope (Carl Zeiss MicroImaging GmbH) using standard filter sets and laser lines and a Plan Apo 63× 1.4 N.A. oil lens. All images within one dataset were taken at fixed intensity settings below saturation.

### Live-cell imaging and quantitative image analysis

HeLa cells stably expressing fluorescently tagged endocytic markers or ESCRTs were grown in MatTek 35 mm glass-bottom dishes (MatTek Corporation). Cells were stimulated for 2 min with 200 ng ml^−1^ EGF-Al647 (E35351, Thermo Fisher Scientific) and then washed with warm Live-Cell Imaging buffer (Invitrogen). In clathrin-depleted cells and the corresponding controls, 600 ng ml^−1^ EGF-Al647 was used instead of 200 ng ml^−1^. Live-cell imaging was performed on an OMX V4 system (DeltaVision OMX Microscope Applied Precision, GE Healthcare) equipped with an Olympus 60× Plan Apochromat 1.42 numerical aperture objective, three cooled PCO.edge sCMOS cameras, a solid-state light source (InsightSSI) and a laser-based autofocus. Environmental control was provided by a heated stage and an objective heater. The 5% CO_2_ and humidity was provided via a CO_2_ mixer (Okolab). Three color live-cell imagings were done in conventional mode at a frame rate of 0.33 Hz. Hardware alignment is done twice a year by GE Healthcare service personal. The *xyz* alignments are controlled regularly and are adjusted if necessary by our core facility staff using bead slides. To guarantee optimal *xy* alignment for every experimental setup, we test the alignment before and after every live-cell imaging session by using the “GE Image Registration slide”. When required, the alignment file was re-calibrated with the help of this slide before entering image files into post-processing for deconvolution and alignment. Acquired images were deconvolved and aligned using Softworx software (Applied Precision, GE Healthcare) and further processed in ImageJ/FIJI. When required, movies were debleached with the ImageJ bleach correction. A custom-made Python script was used for a frame-by-frame co-occurrence analysis. In brief, spots were segmented in all three channels by semi-automated thresholding. Segmented spots were counted as co-occurring when they were less than 5 pixel (i.e., 400 nm) apart. The number of EGF-co-occurring spots in each frame was counted for every movie. The averaged number of co-occurring spots per frame from all movies per condition is displayed as gray values over time. A custom-made Python script was used to manually track individual EGF-positive endosomes in ImageJ and to measure their fluorescence intensity over time. To avoid overlapping fluorescence signals from several microdomains residing on the same endosome, only small EGF-positive vesicles with typically one fluorescent ESCRT spot were tracked.

### High-content microscopy

The Olympus ScanR illumination system with an UPLSAPO 40× objective was used for image acquisition and quantitation of a large number of cells from formaldehyde fixed, immunostained stable cell lines. Identical imaging and analysis settings were applied for all treatments within one experiment. ScanR analysis software was used for background correction and automated image analysis. Fluorescent dots were segmented by the ScanR software, and the total fluorescent intensity of the segmented dots was measured in each cell. The total number of cells was quantified by detection of Hoechst nuclear stain.

### Electron microscopy

HeLa cells were grown on poly-l-lysine-coated sapphire discs. To label newly internalized EGFR following EGF stimulation, cells were first washed with ice-cold PBS and incubated on ice with an antibody recognizing the extracellular part of EGFR (mouse anti-EGFR, Pharmingen). After washing four times with ice-cold PBS, cells were incubated with Protein-A-10 nm gold conjugate (UMC Utrecht Department of Cell Biology) which recognizes the Fc portion of the mouse IgG2b primary antibody. Cell were again washed four times with ice-cold PBS and then stimulated with EGF in warm DMEM for indicated amounts of time before high-pressure freezing was done. Sapphire discs were high-pressure frozen using a Leica HPM100, and freeze substitution was performed as follows: sample carriers designed for sapphire discs were filled with 4 ml of freeze substituent (0.1% (w/v) uranyl acetate in acetone, 1% H_2_O) and placed in a temperature-controlling AFS2 (Leica) equipped with an FPS robot. Freeze substitution occurred at −90 °C for 48 h before the temperature was raised to −45 °C over a time span of 9 h. The samples were kept in the freeze substituent at −45 °C for 5 h before washing 3 times with acetone followed by a temperature increase (5 °C per hour) to −35 °C, and then infiltrated with increasing concentrations of Lowicryl HM20 (10%, 25%, 75%, 4 h each). During the last two steps, temperature was gradually raised to −25 °C before infiltrating 3 times with 100% Lowicryl (10 h each). Subsequent ultraviolet polymerization was initiated for 48 h at −25 °C, and the temperature was then evenly raised to +20 °C (5 °C per hour). Polymerization then continued for another 24 h at 20 °C. Serial sections (~150 nm for counting ILVs in endosomes; 150–250 nm for tomography) were cut on an Ultracut UCT ultramicrotome (Leica, Germany) and collected on formvar-coated mesh grids. Samples were observed at 80 kV in a JEOL-JEM 1230 electron microscope and images were recorded using iTEM software with a Morada camera (Olympus, Germany). Samples that were prepared for tomography were observed in a Thermo ScientificTM TalosTM F200C microscope and image series were taken between −60° and 60° tilt angles with 2° increment. Single-tilt axes series were recorded with a Ceta 16M camera. Tomograms were computed using weighted back projection using the IMOD package. Display and segmentation of tomograms were also performed using IMOD software version 4.9^70^.

Countings were done manually on electron micrographs, and measurements of diameters and length were done in FIJI with the measurement tool. To determine whether gold particles were found in proximitiy to budding profiles, we used a custom-made ImageJ macro to draw a circle with a defined pixel diameter corresponding to 40 nm around each gold particle. When the circle touches the limiting membrane of a budding profile, it was counted as proximal.

### Flow cytometry

Cells were fixed in 70% ethanol and stained with rabbit anti-Histone H3 (phospho S10) antibody (ab5176, Abcam) for 1 h, followed by 30 min of incubation with Alexa Fluor 647 goat anti-rabbit IgG (Jackson). DNA was stained with Hoechst 33242 (1.5 µg ml^−1^). Flow cytometry analysis was performed on LSRII flow cytometer (BD Biosciences) using FACS Diva (BD Biosciences) software and data have been analyzed using FlowJo software. For the percentage of multinucleated cells within the population, cells containing >4N DNA content have been gated.

### EGFR degradation experiments

HeLa cells were stimulated with EGF (50 ng ml^−1^) for 15 min and 60 min in DMEM with 10% fetal calf serum for 1 h before fixation in 3% paraformaldehyde. Cycloheximide (10 µg ml^−1^) was added 60 min before the EGF pulse and was present during the pulse-chase experiment to prevent synthesis of EGFR. The cells were stained with antibodies against EGFR and analyzed by confocal microscopy or lysed and subjected to western blotting as described above. The rescue experiments in Fig. 7a and Supplementary Fig. 3E, F were performed side by side, but split in the figures because of didactic reasons. The coverslips for the IF stainings were made during the same experiments as the western blots and the live-cell imaging as parallel readouts from the same KD experiments.

### Statistical analysis and considerations

The number of individual experiments and the number of cells or endosomes analyzed are indicated in the figure legends. The number of experiments was adapted to the expected effect size and the anticipated consistency between experiments. We tested our datasets for normal distribution and chose an appropriate test accordingly using GraphPad Prism Version 5.01. Unpaired *t*-test was used to test two samples with equal variance, and Mann–Whitney test for samples with unequal variance. For more than two samples, we used one-way analysis of variance (ANOVA) or Kruskal–Wallis test with a suitable post hoc test. All error bars denote mean values ± SD or SEM as indicated in every figure legend; *p < 0.05, **p < 0.01, ***p < 0.001. Samples were not randomized for the experiments. No samples were excluded from the analysis.

### Code availability

Custom-made image analysis scripts are available on https://github.com/koschink/Wenzel_et_al_2018.

## Electronic supplementary material

Supplementary Information

Peer Review File

Description of Additional Supplementary Files

Supplementary Movie 1

Supplementary Movie 2

## Data Availability

Additional raw data that support the findings of this study are available from the corresponding author upon reasonable request.
