# Supplementary material for: Concerted ESCRT and clathrin recruitment waves define the timing and morphology of intraluminal vesicle formation
Source: Nat Commun. 2018 Jul 26;9:2932. doi: 10.1038/s41467-018-05345-8 (PMC6062606; doi:10.1038/s41467-018-05345-8)
Supplement: Supplementary file 1 — Supplementary Information [file 41467_2018_5345_MOESM1_ESM.pdf]

**Supplementary Information**

# Concerted ESCRT and Clathrin recruitment waves define the timing and morphology of intraluminal vesicle formation

**Wenzel et al. 2018**

## Supplementary Fig. 1

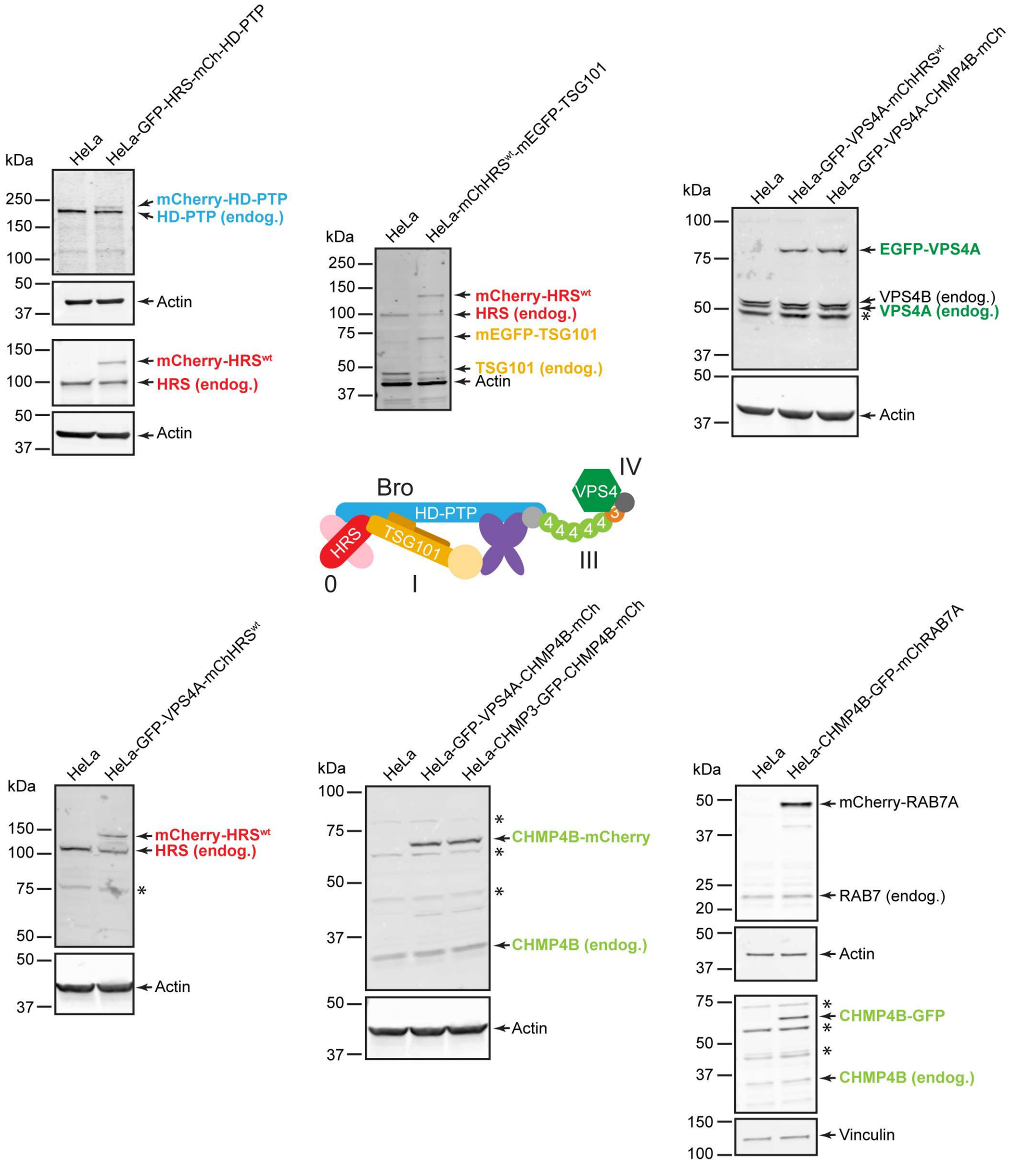


**Supplementary Fig. 1. Stable cell lines express tagged proteins at close to endogenous levels.** Western blots showing the expression of fluorescently tagged ESCRT proteins and RAB7 as indicated and in relation to their endogenous counterparts. Representatives of the ESCRT subcomplexes are color-coded according to the schematic. We could not investigate CHMP3 expression levels due to lack of a working antibody. Antibody specificity was verified by depletion experiments to identify the correct endogenous band. Asterisks indicate unspecific bands.

## Supplementary Fig. 2

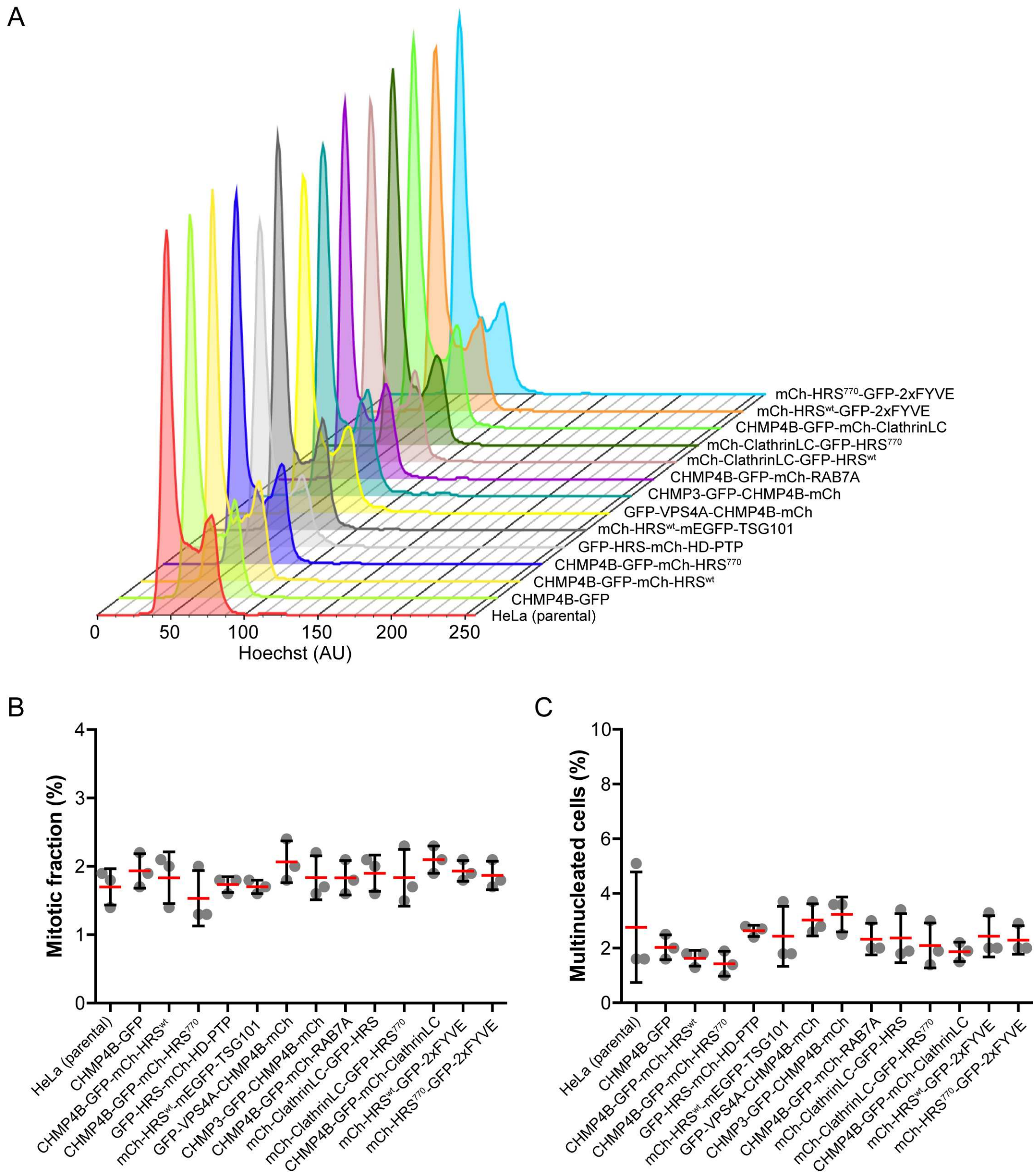


**Supplementary Fig. 2. Flow cytometry analysis shows no change in proliferation or multinucleation in cell lines stably expressing tagged ESCRT proteins.**

Parental HeLa cells or cell lines stably expressing ESCRT proteins as indicated were fixed and stained with a phospho-Histone-H3 antibody and incubated with Hoechst dye before they were analyzed by flow cytometry.

(A) Analysis of DNA profiles show similar cell cycle distributions and none of the cell lines show any signs of cell cycle aberrations. Shown is one representative of three independent experiments.

(B) Cell proliferation was further investigated with phospho-Histone-H3 stainings. None of the cell lines show abnormal levels of mitotic cells. Kruskal-Wallis test: not significant. Data presented as mean +/- SD from three independent experiments.

(C) Multinucleation was assessed by gating for DNA contents larger than 4N. No abnormalities were observed. Kruskal-Wallis test: not significant. Data presented as mean +/- SD from three independent experiments.

## Supplementary Fig. 3

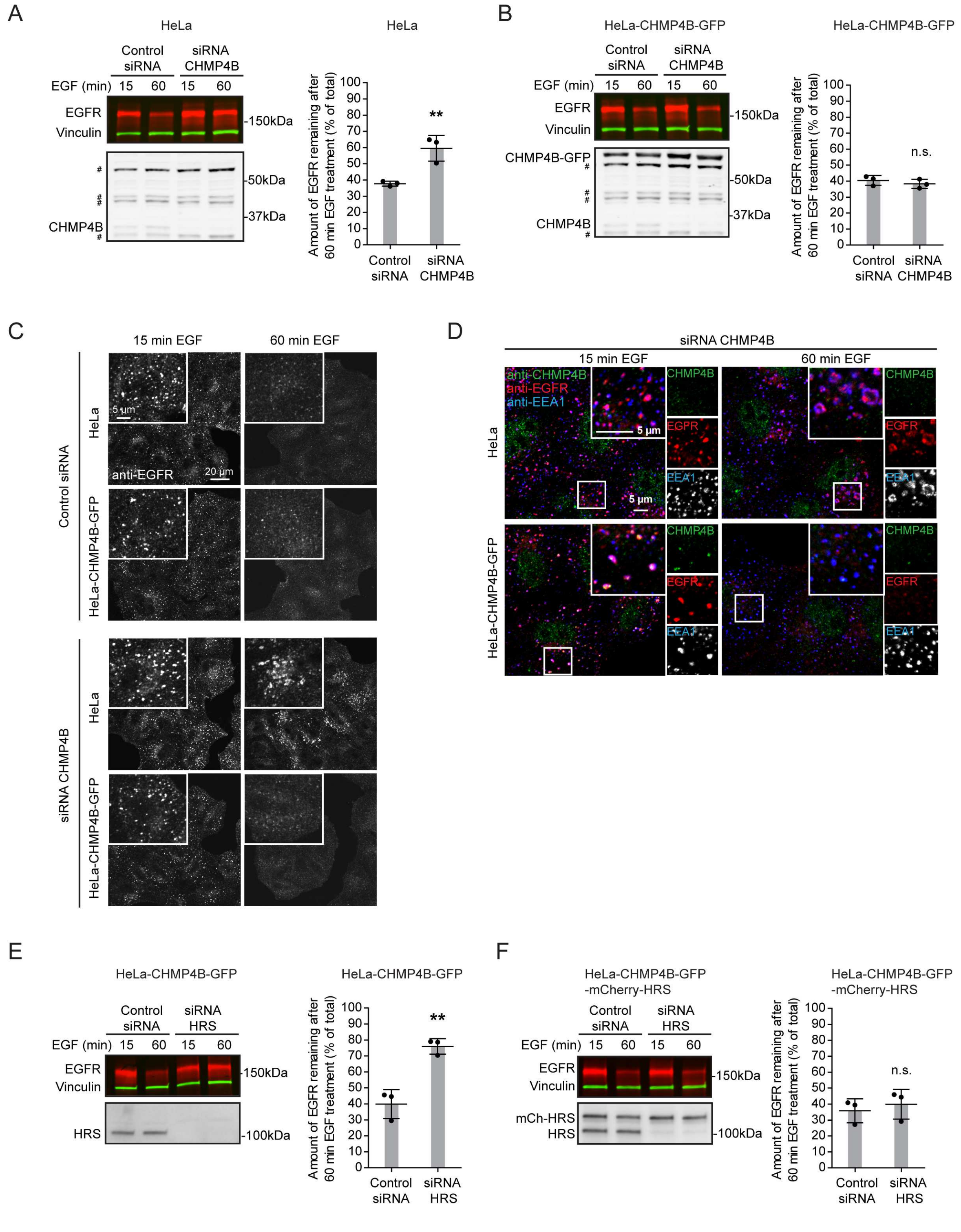

**Supplementary Fig. 3. HeLa cell lines stably expressing fluorescently tagged ESCRT proteins are fully functional in degrading EGFR**

(A) siRNA-mediated depletion of endogenous CHMP4B leads to impaired degradation of EGFR after EGF stimulation when compared to control as analyzed by quantitative WB analysis. # indicates unspecific bands. Data are represented as mean +/- SD. t-test, ** $p<0.01$. n=3 experiments.

(B) The impaired EGFR degradation in cells depleted for endogenous CHMP4B is rescued in HeLa-CHMP4B-GFP cells stably expressing siRNA resistant CHMP4B-GFP. # indicates unspecific bands. Data are represented as mean +/- SD. t-test, n.s. not statistically significant. n=3 experiments.

(C) Immunofluorescence staining of EGFR 15 and 60 min after EGF stimulation shows efficient degradation of the receptors after 60 min in the parental HeLa cells and in HeLa-CHMP4B-GFP cells, indicating that expression of CHMP4B-GFP does not impair EGFR degradation. Depletion of endogenous CHMP4B leads to a severely impaired EGFR degradation, which is rescued in the cell line stably expressing CHMP4B-GFP. Data is representative of 2 independent confocal microscopy experiments.

(D) Immunofluorescence staining of EGFR and the early endocytic marker EEA1 shows accumulation of EGFR in early endocytic compartments when CHMP4B is depleted. This accumulation is rescued in the CHMP4B-GFP expressing cell line, which degrades EGFR. Note an unspecific signal for CHMP4B in the nucleus, which is not reduced upon knockdown, while the endosomal CHMP4B signal disappears in siRNA CHMP4B treated cells. CHMP4B-GFP appears to be strongly recruited to endosomes 15 min after EGF stimulation and less after 60 min. Data is representative of 2 independent confocal microscopy experiments.

(E) siRNA-mediated depletion of endogenous HRS leads to impaired degradation of EGFR after EGF stimulation when compared to control as analyzed by quantitative WB analysis. Data are represented as mean +/- SD. t-test, ** $p<0.01$. n=3 experiments.

(F) The impaired EGFR degradation in cells depleted for endogenous HRS is rescued in HeLa-CHMP4B-GFP-mCherry-HRS cells stably expressing siRNA resistant mCherry-HRS. Data are represented as mean +/- SD. t-test, n.s. not statistically significant. n=3 experiments.

# Supplementary Fig. 4

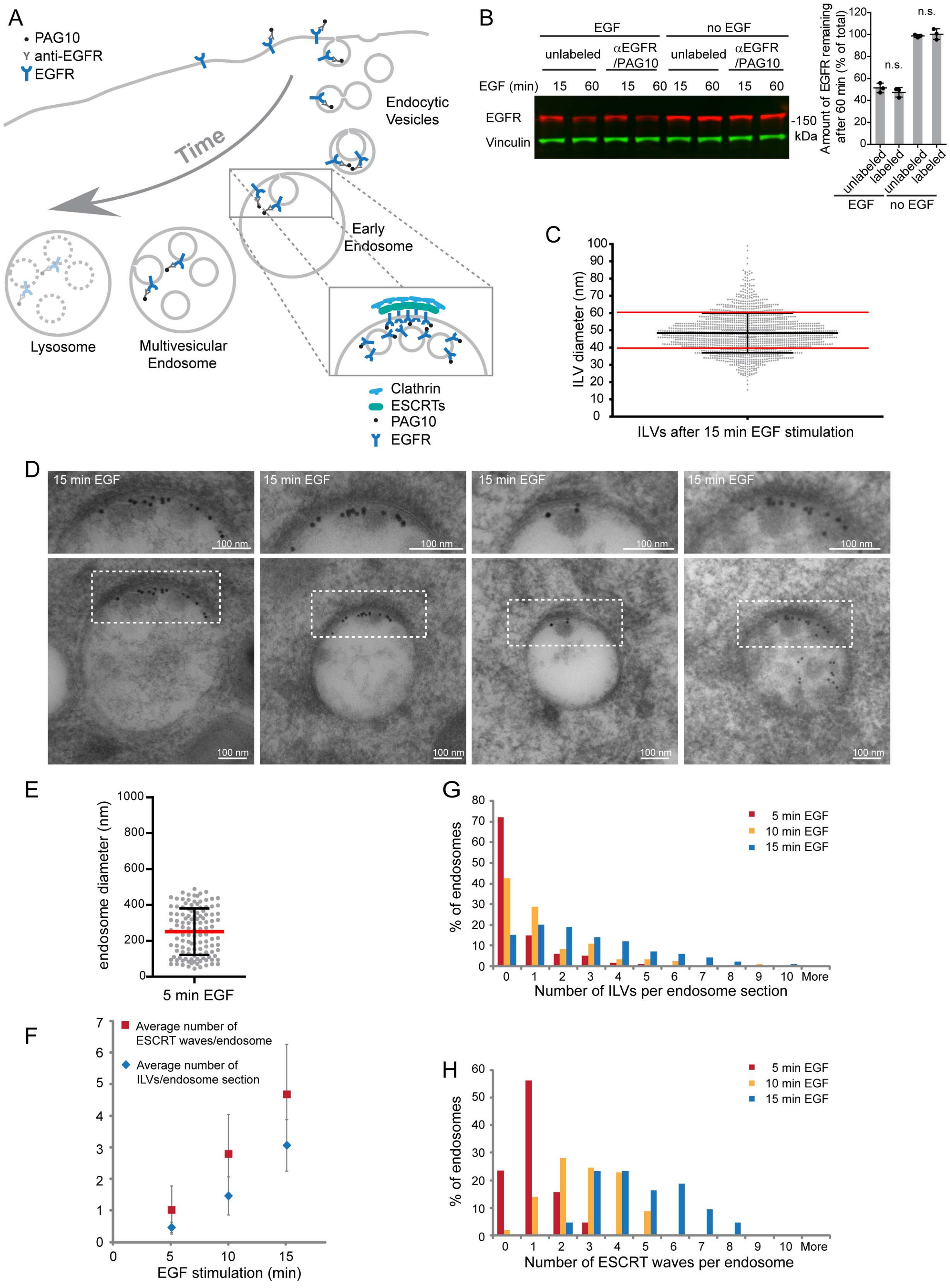

**Supplementary Fig. 4. Correlation between the number of ESCRT waves and ILV formation.**

(A) Experimental setup for EM experiments: HeLa cells were incubated with an antibody targeting the extracellular part of EGFR, followed by a labeling with protein-A-gold (10 nm) (PAG10). Labeling was done on ice to avoid internalization of EGFR during the labeling procedure. Cells were then stimulated with 50 ng $ml^{-1}$ EGF for different time points before high-pressure freezing and sample preparation for EM. Gold particles serve to identify newly internalized EGFR-containing endosomes. Inset illustrates the topology of EGFR within an endosomal microdomain consisting of cargo, ESCRTs and clathrin. Note that since ILVs are typically found directly under the limiting membrane of the MVE, it is often difficult to distinguish whether the gold particles are associated with receptors in the limiting membrane or with receptors associated with internalized ILVs. In addition, ILV associated gold particles can be located above or below the section and therefore not visible.

(B) Quantitative WB analysis shows that EGFR degradation is not affected by anti-EGFR/PAG10 labeling on ice and anti-EGFR/PAG10 labeling does not lead to EGF-stimulation independent EGFR downregulation due to possible crosslinking. Data represents mean +/- SD of 3 independent experiments. t-test, n.s. not statistically significant.

(C) ILV size distribution from >1500 ILV measurements from 15 min EGF stimulation EM experiments. Indicated are diameters of 40 and 60 nm (red lines). ILVs between 40 and 60 nm diameter were considered typical ESCRT-dependent ILVs and used for counting the number of ILVs per endosome section.

(D) Gallery of electron micrographs showing different examples of gold labeled MVEs. Note that ILVs are usually found in proximity to gold labeled EGFR and the endosomal clathrin coat.

(E) Endosome diameters of gold labeled endosomes were measured after 5 min of EGF stimulation. At least 100 endosomes were measured from 3 independent samples. Dot plot indicates mean +/- SD.

(F) Quantification of the average number of ESCRT waves counted from live-cell microscopy tracks after 5, 10 and 15 min of EGF stimulation and correlated to the average number of ILVs observed per endosome section in electron microscopy after 5, 10 and 15 min of EGF stimulation. Error bars indicate SD. The imaging data is averaged from 64, 57 and 43 fluorescence intensity measurements, respectively, which stem from 21 independent live-cell imaging experiments. For the EM analysis at least 100 gold labeled endosomes were analyzed per time point from 3 independent samples.

(G) Histogram showing the percentage of endosomes with the indicated number of ILVs per endosome section after 5, 10 and 15 min of EGF stimulation. Same dataset as used in Fig. 3c and Supplementary Fig. 4F.

(H) Histogram showing the percentage of endosomes with the indicated number of ESCRT waves counted from live-cell microscopy tracks after 5, 10 and 15 min of EGF stimulation. Same dataset as used in Fig. 3c and Supplementary Fig. 4F.

## Supplementary Fig. 5

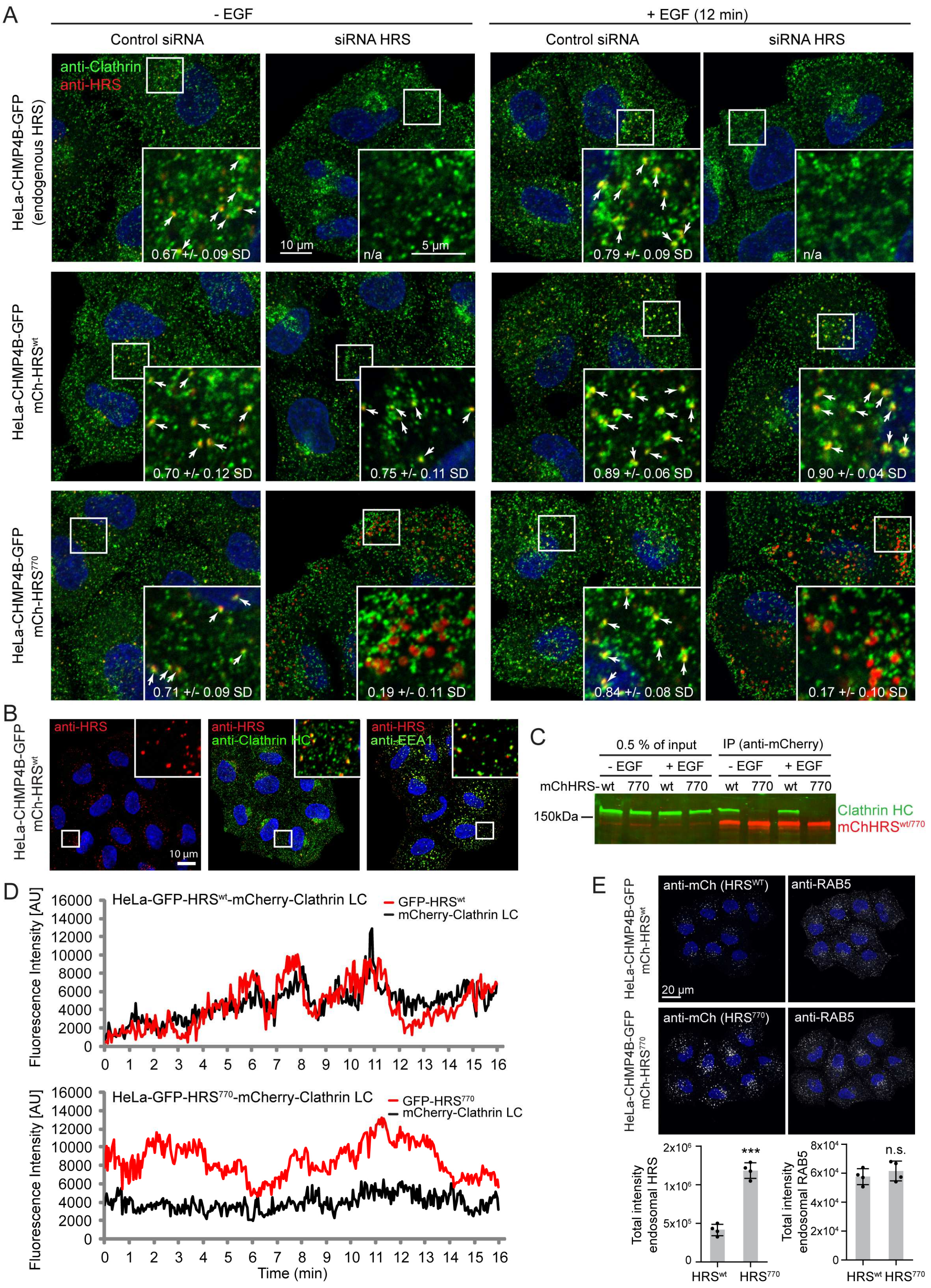

**Supplementary Fig. 5. Characterization of the HRS$^{770}$ mutant.**

(A) The indicated cell lines were depleted for endogenous HRS as shown in Fig. 5b, EGF stimulated or not and then immunostained for clathrin heavy chain (green) and HRS (red). Numbers indicate overlap of HRS with clathrin (MCC). n/a, not applicable. Arrows indicate HRS-positive endosomes colocalizing with clathrin. Note that in contrast to mCherry-HRS$^{wt}$, mCherry-HRS$^{770}$ does not recruit clathrin to endosomes when cells are depleted for endogenous HRS. Data is generated from 7-10 confocal microscopy pictures comprising > 20 cells per condition.
(B) Control experiment for the experiment shown in Supplementary Fig. 5A and Fig. 4d: Under the chosen imaging conditions, the CHMP4B-GFP signal is not visible and therefore it was possible to detect endogenous clathrin HC with an Al488 secondary antibody. Further we confirm that the HRS signal overlaps with an endosomal marker (EEA1).
(C) Co-immunoprecipitation experiment to verify biochemically that the interaction of HRS with clathrin is abolished upon deletion of the clathrin binding box in HRS$^{770}$ in EGF stimulated and unstimulated cells. Western blot is representative from 3 independent experiments.
(D) HeLa cells stably expressing mCherry-Clathrin LC in combination with EGFP-HRS$^{wt}$ or –HRS$^{770}$ were depleted for endogenous HRS. Fluorescence intensity profiles of tracked EGF-positive endosomes over time verify that in contrast to HRS$^{wt}$, the hyperstabilized HRS$^{770}$ does not recruit mCherry-Clathrin LC. The tracks are representative examples of 7 (HRS$^{wt}$) and 6 (HRS$^{770}$) tracks from 3 independent experiments per condition.
(E) HeLa cells stably expressing mCherry-HRS$^{wt}$ or –HRS$^{770}$ were depleted for endogenous HRS. Immunostaining for mCherry-HRS and endogenous RAB5 shows that HRS fluorescence intensity is increased in the HRS$^{770}$ expressing cells compared to HRS$^{wt}$, while RAB5 intensities are unaffected. Quantification of fluorescence intensities is done by high-content microscopy of >1500 cells per condition from 4 experiments.

## Supplementary Fig. 6

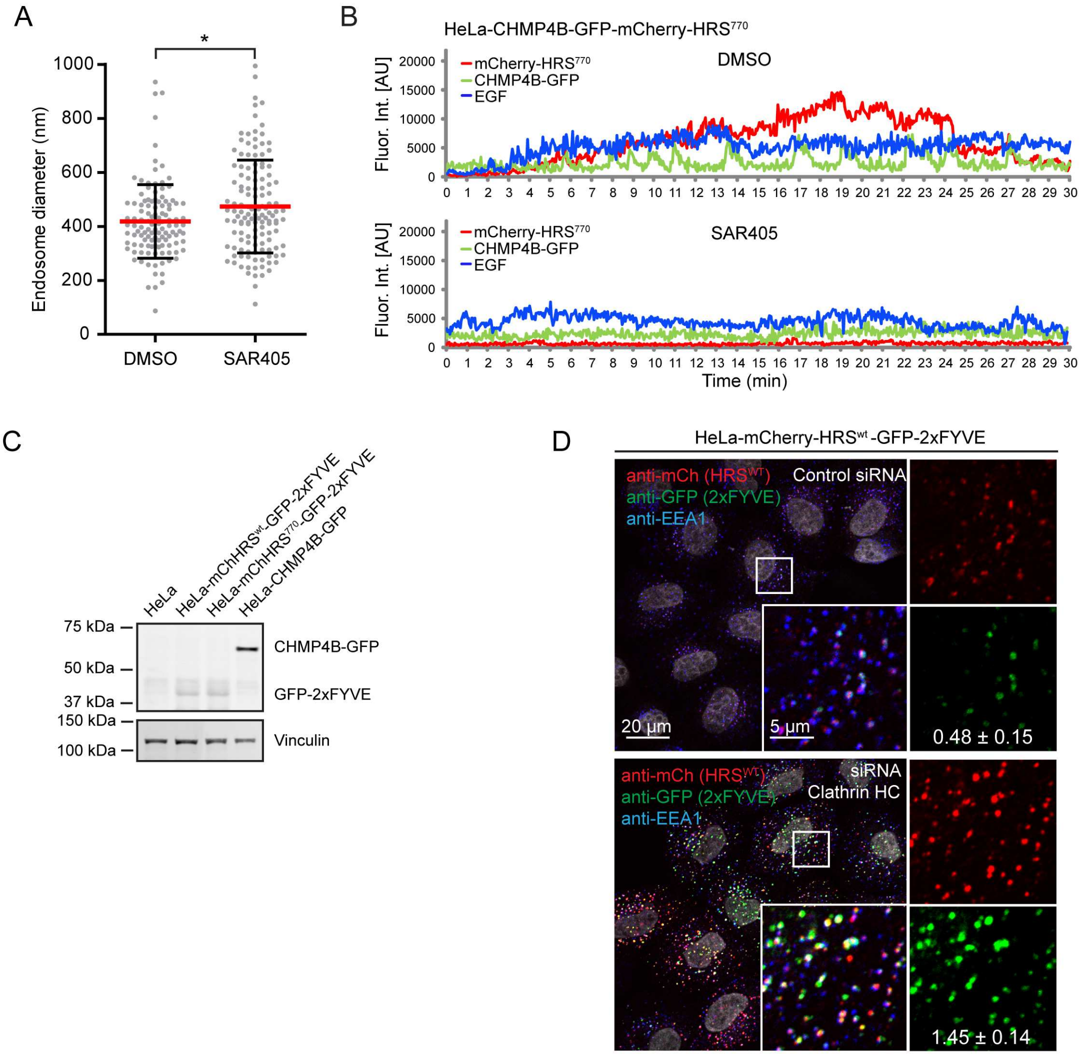


**Supplementary Fig. 6. Initial recruitment of HRS$^{770}$ is dependent on PtdIns3P and clathrin depletion affects PtdIns3P levels on endosomes.**

(A) Diameter of gold labeled endosomes after 15 min EGF stimulation in DMSO or SAR405 treated cells was measured from electron micrographs (same dataset as Fig. 6f). Data is presented as dot plot with mean +/- SD. ≥100 endosomes were analyzed per condition from 2 independent experiments. Mann-Whitney test, * $p<0.05$.

(B) Fluorescence intensity profiles over time of two representative EGF-positive endosomes from HeLa-CHMP4B-GFP-mCherry-HRS$^{770}$ cells depleted for endogenous HRS show hyperrecruitment of mCherry-HRS$^{770}$ in the presence of DMSO (upper panel) and lack of recruitment of mCherry-HRS$^{770}$ and CHMP4B to newly formed endocytic vesicles in the presence of SAR405 (lower panel).

(C) Western blot (anti-GFP) showing the expression level of GFP-2xFYVE in HeLa-mCherry-HRS$^{wt}$-GFP-2xFYVE and HeLa-mCherry-HRS$^{770}$-GFP-2xFYVE stable cell lines. To give an indication of the level of overexpression of the 2xFYVE probe, a lysate of HeLa-CHMP4B-GFP cells is included.

(D) HeLa cells stably expressing the PtdIns3P probe GFP-2xFYVE together with mCherry-HRS$^{wt}$ were depleted for clathrin heavy chain or control siRNA transfected. Clathrin depleted cells show increased levels of GFP-2xFYVE on endosomes when compared to control cells. Total fluorescence intensities of endosomal GFP-2xFYVE per cell were quantified by high-content microscopy and are indicated as AU (x10$^{5}$) +/- SD. Data is quantified from >2500 cells per condition from 4 independent experiments. GFP-2xFYVE intensity (control versus siRNA clathrin HC): t-test, $p<0.001$.

## Supplementary Fig. 7

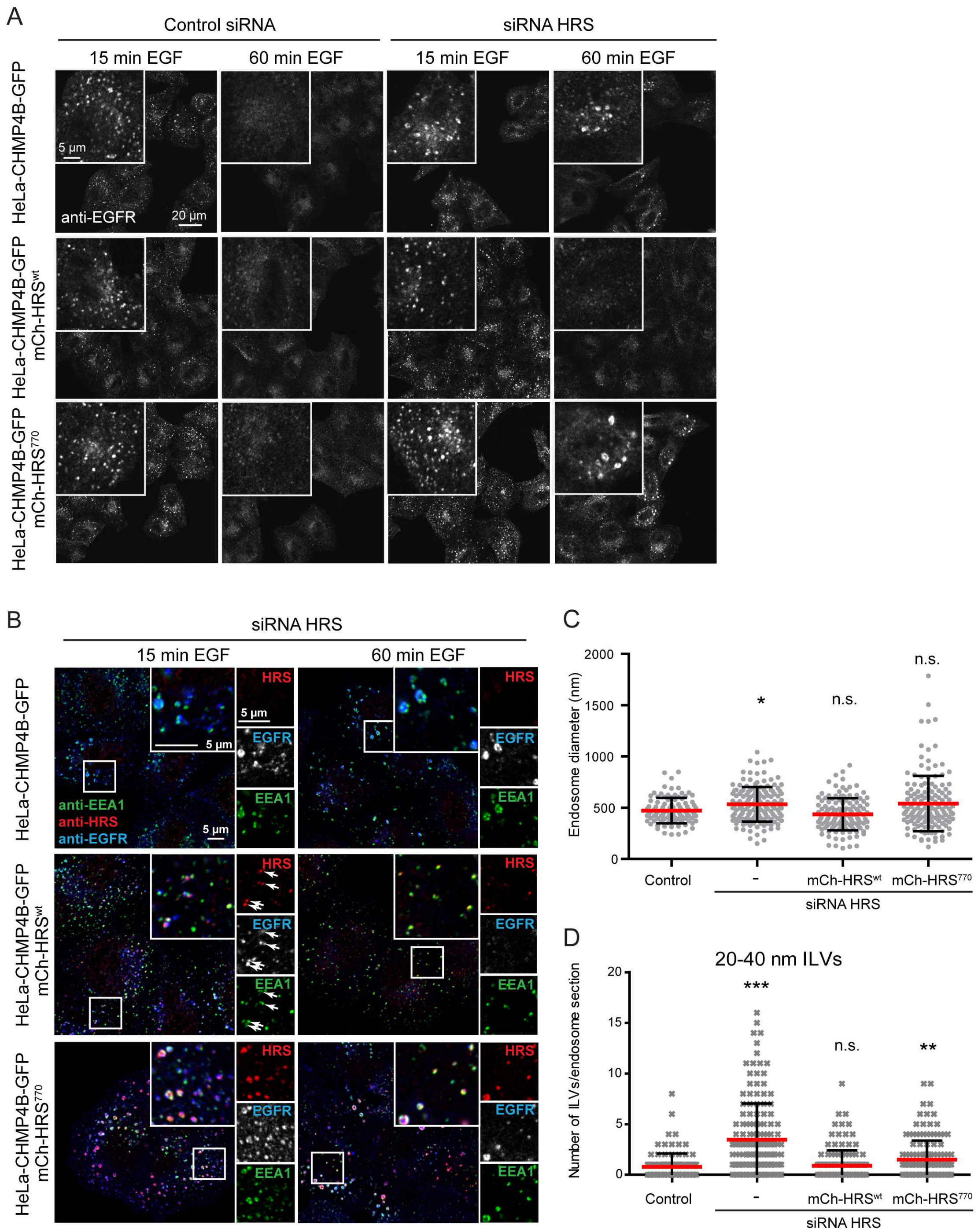

**Supplementary Fig. 7. Rescue experiments reveal the importance of clathrin recruitment to endosomes for cargo degradation and ILV formation.**

(A) Immunofluorescence staining of EGFR receptors 15 and 60 min after EGF stimulation shows efficient degradation of the receptors after 60 min in HeLa-CHMP4B-GFP cells, with or without expression of mCherry-HRS$^{wt}$ or –HRS$^{770}$, indicating that expression of mCherry-HRS$^{wt}$ or –HRS$^{770}$ does not impair EGFR degradation. Depletion of endogenous HRS (siRNA HRS) leads to a severely impaired EGFR degradation, which is rescued in the cell line stably expressing mCherry-HRS$^{wt}$, but not mCherry-HRS$^{770}$. Data is representative of 2 independent confocal microscopy experiments.

(B) Immunofluorescence staining of EGFR, HRS and the early endocytic marker EEA1 shows accumulation of EGFR in early endocytic compartments when HRS is depleted. This accumulation is rescued in the mCherry-HRS$^{wt}$ expressing cell line, which degrades EGFR, while mCherry-HRS$^{770}$ shows a strong accumulation of EGFR at EEA1 positive endosomes. Data is representative of 2 independent confocal microscopy experiments.

(C) Diameter of gold labeled endosomes after 15 min EGF stimulation as measured from electron micrographs (same dataset as Fig. 7c,d). Data is presented as dot plot with mean +/- SD. More than 100 endosomes were analyzed per condition from at least 3 different samples. Kruskal-Wallis test, $p<0.001$. Dunn’s multiple comparison test: *$p<0.05$; n.s. not statistically significant.

(D) Quantification of the number of small (<40 nm), presumably ESCRT-independently formed, ILVs per endosome section (same dataset as Fig. 7c,d). Data is presented as dot plot with mean +/- SD. Kruskal-Wallis test, $p<0.001$. Dunn’s multiple comparison test: **$p<0.01$; ***$p<0.001$; n.s. not statistically significant.

## Supplementary Fig. 8

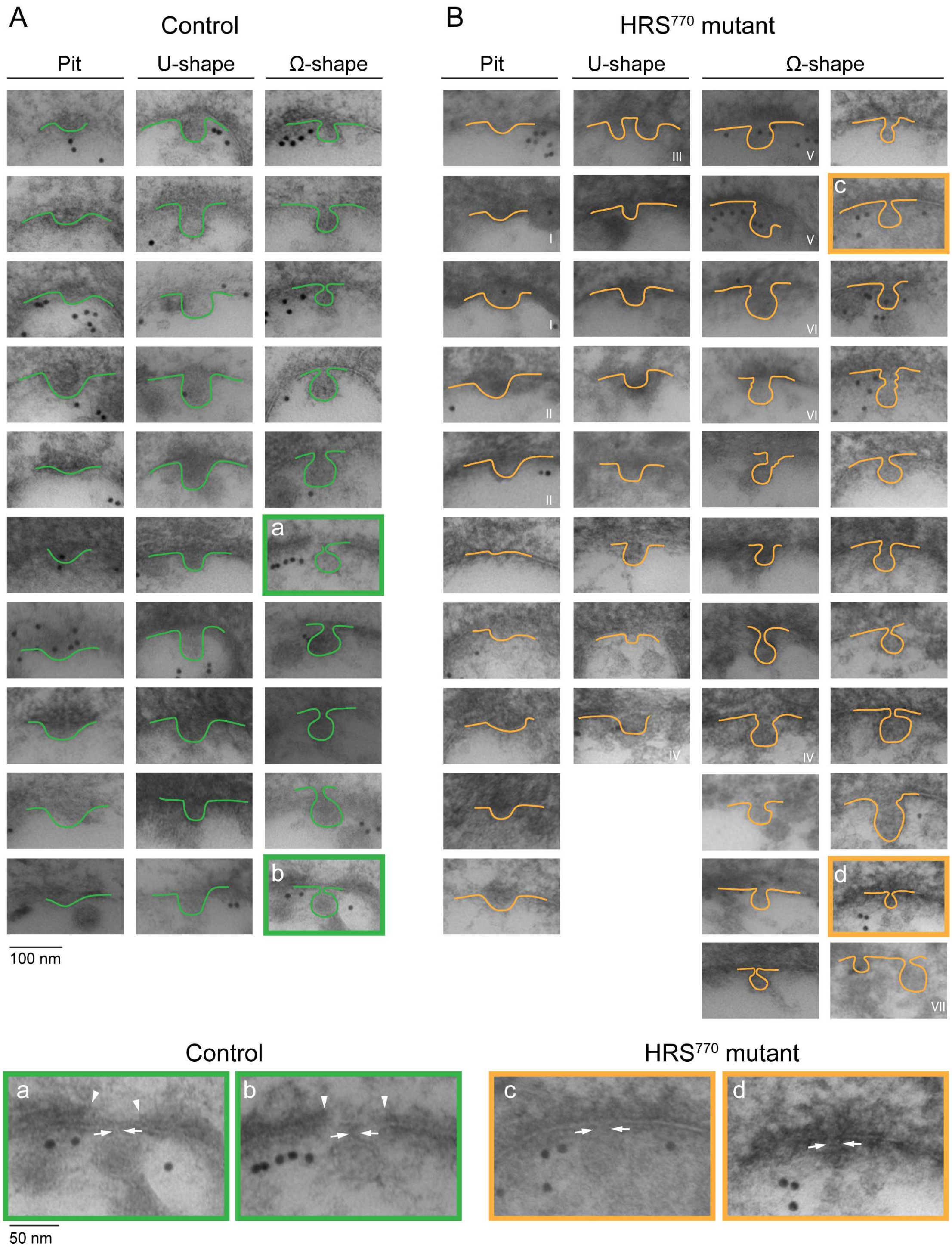

**Supplementary Fig. 8. Gallery of ILV budding profiles.**

(A) Gallery of electron micrographs showing examples of newly forming ILVs. Budding profiles were taken from HeLa cells treated for 5, 10 or 15 min with EGF, or for 15 min with EGF in the presence of DMSO, or for 15 min with EGF in siHRS/HRS$^{wt}$ rescue experiments. Two omega-shaped budding profiles are magnified. Note the absence of the EGFR/HRS/clathrin coat directly above the almost abscised ILV. Arrowheads depict the boundaries of the EGFR/HRS/clathrin coat, arrows the neck of the budding profile.

(B) Gallery of electron micrographs showing examples of newly forming ILVs from HRS$^{770}$ expressing cells. Roman numerals in images indicate budding profiles found on the same endosome section. Two omega-shaped budding profiles from HRS$^{770}$ mutant expressing cells are magnified. Arrows indicate the neck of the budding profile. Note that the putative EGFR/HRS$^{770}$ coat appears homogenous and solid directly above the forming ILV.

**Supplementary Fig. 9**

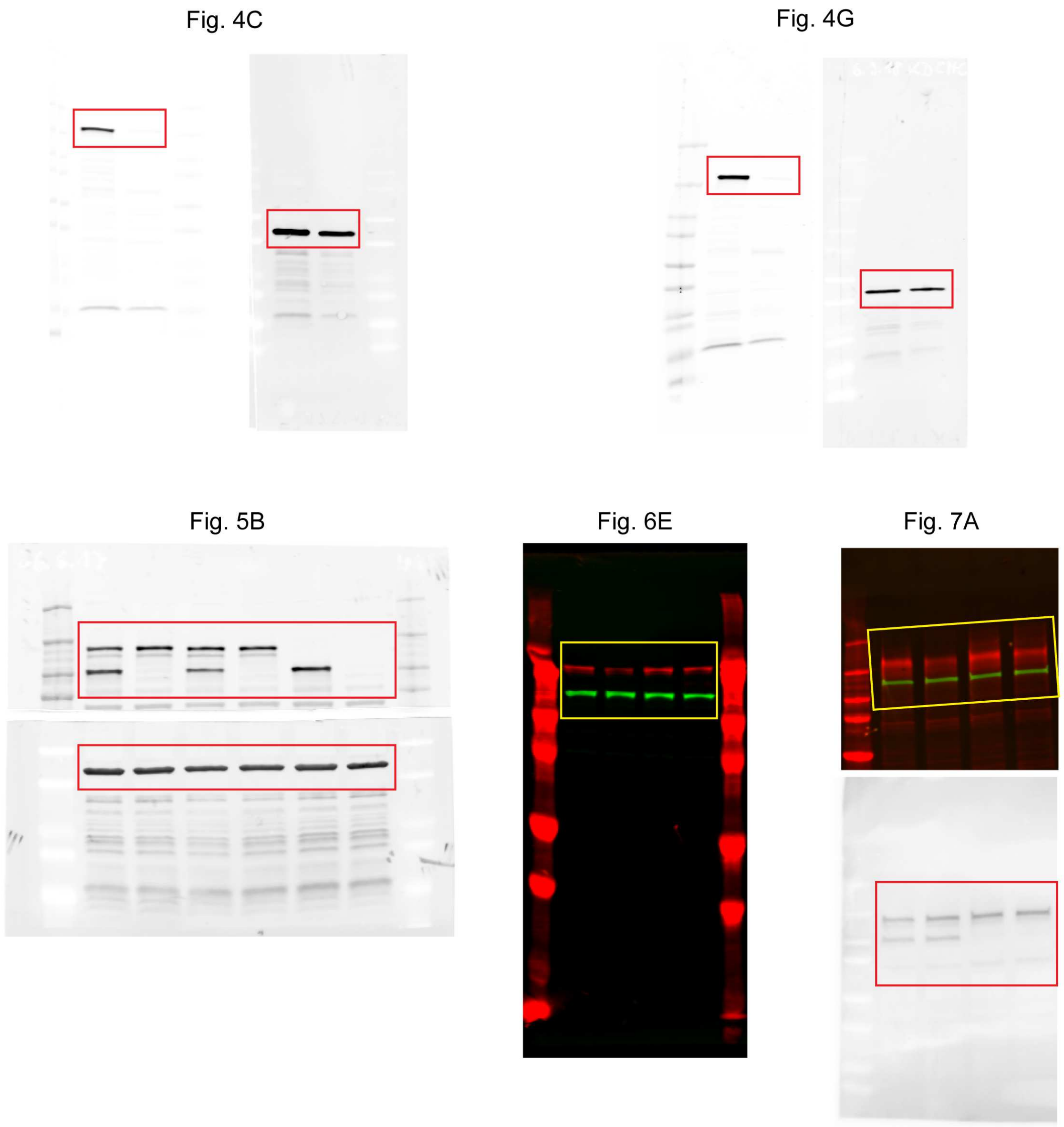


**Supplementary Fig. 9. Uncropped Western blots.**
Displayed are uncropped membranes from the main figures as indicated.

## Supplementary Fig. 10

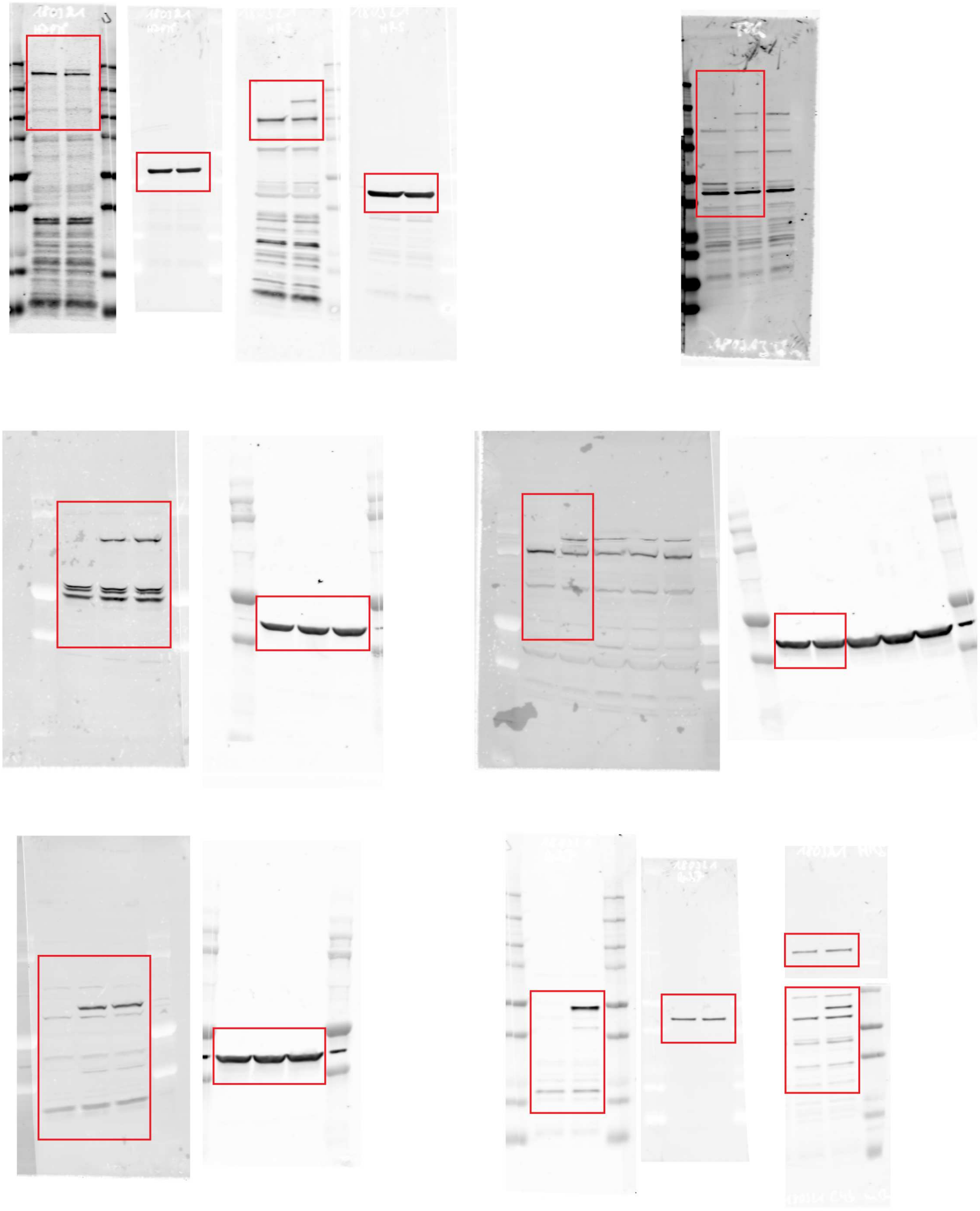

**Supplementary Fig. 10. Uncropped Western blots.**
Displayed are uncropped membranes from Supplementary Fig. 1.

## Supplementary Fig. 11

Supplementary Fig. 3A, B

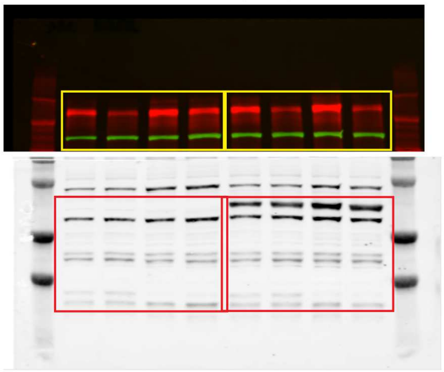

Supplementary Fig. 3E

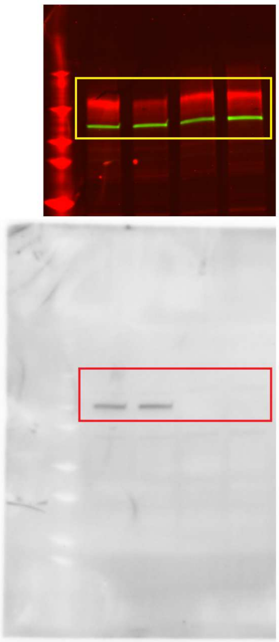

Supplementary Fig. 3F

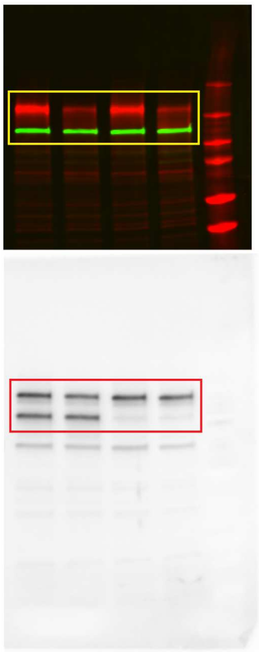

Supplementary Fig. 4B

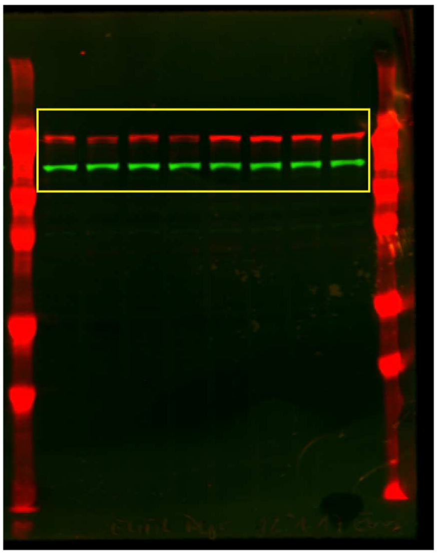

Supplementary Fig. 5C

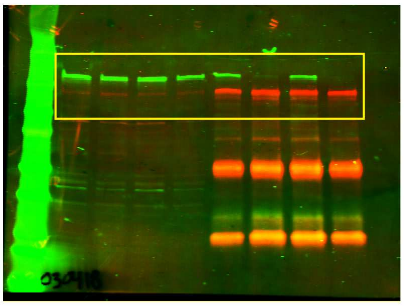

Supplementary Fig. 6C

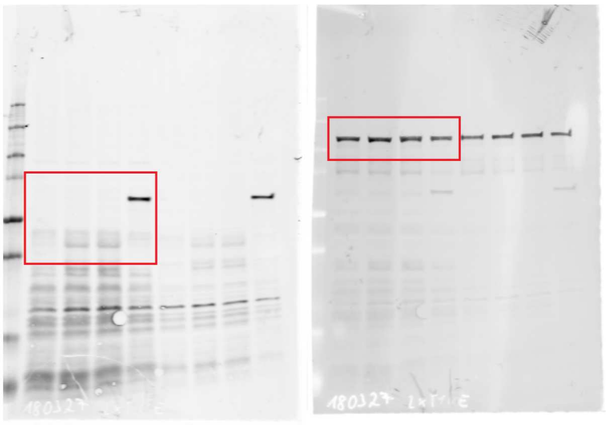

**Supplementary Fig. 11. Uncropped Western blots.**
Displayed are uncropped membranes from the Supplementary Figures as indicated.
