# Supplementary material for: Concerted ESCRT and clathrin recruitment waves define the timing and morphology of intraluminal vesicle formation
Source: Nat Commun. 2018 Jul 26;9:2932. doi: 10.1038/s41467-018-05345-8 (PMC6062606; doi:10.1038/s41467-018-05345-8)
Supplement: Supplementary file 3 — Description of Additional Supplementary Files [file 41467_2018_5345_MOESM3_ESM.docx]

**Description of Additional Supplementary Files**

File Name: Supplementary Movie 1

Description: Movie illustrating a representative live cell imaging experiment. HeLa-CHMP4B-GFP-mCherry-HRS expressing cells were stimulated with EGF-Al647 (blue) before starting the time-lapse imaging experiment (see Fig. 2A). CHMP4B-GFP, green, mCherry-HRS, red. Highlighted and enlarged is the tracked endosome corresponding to Fig. 2C.

File Name: Supplementary Movie 2

Description: Illustration of the fluorescence intensity measurements on individually tracked endosomes.
