# Supplementary material for: Concerted ESCRT and clathrin recruitment waves define the timing and morphology of intraluminal vesicle formation
Source: Nat Commun. 2018 Jul 26;9:2932. doi: 10.1038/s41467-018-05345-8 (PMC6062606; doi:10.1038/s41467-018-05345-8)
Supplement: Supplementary file 2 — Peer Review File [file 41467_2018_5345_MOESM2_ESM.pdf]

Reviewers' comments:

Reviewer #1 (Remarks to the Author):

Review of "Concerted ESCRT and Clathrin recruitment waves define the timing and morphology of intraluminal vesicle formation".

The authors set out to investigate the timing of ESCRT and clathrin recruitment during the formation of intraluminal vesicles involved in receptor signaling degradation. This is an important research topic and the gained insight could help to understand the mechanism of how ESCRTs support scission. However, this reviewer believes that there are some potentially serious problems that need to be addressed in both how the experiments were conducted or interpreted that currently restrain the ability to evaluate significance. In particular, this would be a much strong work if the authors could better characterize the cell lines created for this work, redo certain experiments (as explained further below) and perform additional experiments (suggestions given below).

Major issues:

1. It is claimed that the CHMP4B cell line expresses CHMP4B-GFP at low levels. However, in the Western Blot (Suppl. Figure S1 B) there is much greater than 20-fold overexpression of the GFP-tagged version compared to the endogenous version. All other cell lines generated for this study do not seem to have been characterized (there is no characterization described other than stating that "additional stable HeLa cell lines with close to endogenous expression"), no WBs were shown of the expression levels of the stably expressed proteins, no cell behavior (such as percentage of multinucleated cells or growth rates compared to parental strains) was tested. This is a major problem since it is well established that even modest over-expression of ESCRTs inhibits the processes they are involved in. There are also numerous claims that overexpressed ESCRTs can been found in many non-native locations. Thus, assuming a 20x overexpression (and in the provided Western Blot, it looks greater), even if the CHMP4B-GFP is functional it may be that 5% of what is expressed is sufficient to complement what is lost by the siRNA, and that is properly localized. However, the remaining 95% is mislocalized. The methods section does not give any detail on how these cell lines were generated or tested, other than the authors thanking the FACS facility for help for sorting stable cell lines, so possibly clonal cell lines were established? This is a major concern and needs to be addressed before any of the results gained from experiments with these cell lines can be properly evaluated.

2. The described limit for co-localization of 5 pixels (400 nm) seems too high. Since the structures that are tested for co-localization are, at least according to the figures shown, much smaller than 400 nm, this reviewer believes that the limit must be set to smaller values. Thus, this reviewer is not convinced that the markers tested do indeed colocalize as stated in the manuscript.

3. The involvement of clathrin in this process is an interesting and potentially important story. Obviously, any disruption of clathrin will affect many steps of membrane transport in the endocytic system and thus affect sorting and targeting in the multivesicular bodies. Thus, a first step is to nail down the correlation of co-localization and then explore if there is a causal link. In Suppl. Figure 4 A to test for co-localization it needs to be quantified. Additionally, what is the relationship/localization of HRS relative to clathrin in cells not treated with siRNA? This is important to be able to judge the rescue experiments. To this reviewer the image shown for the HRS770 rescue looks as if there are a few spots with co-localization. The evidence for a link would be stronger if this experiment was done with EGF stimulation to test if clathrin is co-localizing to endosomes with the mutant HRS upon stimulation. This would help to establish clathrin as the cause of the ILV phenotype when HRS770 is used. If clathrin still gets recruited, then the given explanation of clathrin causing the failure to form ILVs of the correct size and shape would be wrong. A second problem is that it is not clear which label on the secondary antibody was used to

generate these images? Is it compatible with imaging GFP or in this case, making sure that GFP does not contribute to the signal?

4. Please explain the mCherry experiment in Figure 5E, this reviewer cannot find any reference to it.

5. In general, the results with HRS with deletion of its clathrin-interacting domain (HRS770) are presented in a way as if this proves causality for the failure to generate ILVs. While this is a nice demonstration, this reviewer believes that more experiments are necessary to be able to make this claim. Some are already described above, but there should also live cell imaging experiments performed in cells where the behavior of clathrin is imaged together with HRS or HRS770 and CHMP4B in cells that were treated with EGF and in non-stimulated cells.

6. In my opinion, the results do not support the statement on page 8 that "This indicates that clathrin regulates the dissociation of ESCRT-0, but not ESCRT-III.", it was only shown that clathrin is involved in the dissociation of HRS.

7. Page 10, Phase 1 paragraph, line 9: It is hard to follow why the authors suggest "that clathrin alters the membrane tension required for initiating pit formation." What results is this based on? Clathrin by itself cannot interact with the membrane bilayer, it interacts through various adapters, so how is membrane tension affected? The authors use this argument to explain how absence of clathrin causes the failure to form ILVs, so this is an important and should be explained how the authors believe that this occurs. Related to this, the authors state on page 11, third paragraph, line 4, that "clathrin indirectly regulates" the dissociation of HRS from endosomes upon its phosphorylation, but no mechanism is suggested. Do the authors believe that auxilin and synaptojanin (mentioned further below in the manuscript) are involved in the endosomal setting too? That can be tested via imaging.

8. The summary paragraph states that the authors have "uncovered novel functions for clathrin in these processes", while this reviewer believes that the authors only showed that clathrin is present, not necessarily that it functions in ILV generation.

Minor issues:

1. Details seem to be missing in how the fixed cell experiments have been performed: If live "cells grown on coverslips were permeabilized with PEM buffer", does this mean they were treated with detergent for 10min and then fixed? This raises the concern that a lot of cytosolic signal was lost before fixation possibly invalidating the results shown after such a prolonged permeabilization. The immunofluorescence images might not be representative for the protein distribution in live cells. No detergent was mentioned in the description of the PEM buffer. Can the PEM Buffer permeabilize the membrane or were other chemicals used that are not mentioned in the methods? Some clarification is needed in the methods section.

2. In figure 3 D the fluorescence intensity signal seems very high for signal from single endosomes (peaks around 20,000 a.u.) in live-cell imaging over 30 minutes. What laser power was used? At that laser power, what is the time course for bleaching? Was the signal amplified via applying a camera gain? Then this should be described in the methods section.

3. Please specify which sCMOS cameras were used. How were the three cameras aligned and how was the registration tested?

4. Two references in the Methods section of "Immunostaining, antibodies and reagents" were not formatted, the second one is altogether missing in the list of references, the first one could be ref 3 or ref 27.

5. The objective used on the Zeiss LSM microscope was misspelled: 631.4NA should be replaced by 63x 1.4 N.A.

6. Page 7, last paragraph, sixth line: the wrong figure number was given, it should be 4D instead if 3D.

7. Figure 4 D: the y axis has no label

8. Suppl. Figures S1: please explain the * and ** on the WBs on the left of A and B.

9. The reference for the antibody to Chmp is not formatted properly.

Reviewer #2 (Remarks to the Author):

Wenzel et al in this study have used live imaging to study the dynamics of ESCRT and clathrin recruitment during intralumenal vesicle (ILV) formation at endosomes. Using stable cell lines that mildly overexpress fluorescent protein tagged variants of various ESCRT proteins or clathrin they show that the ESCRT-O component Hrs is gradually recruited over minutes whereas the late-acting ESCRT-III factor CHMP4B displays transient waves of recruitment before it dissociates together with Hrs, likely reflecting the formation of a single ILV as suggested by EM experiments. Interestingly, clathrin, which was shown before to form an Hrs-dependent coat on early endosomes regulates ILV formation and sorting by modulating ILV bud size and by facilitating concerted Hrs removal. These data are taken to propose a new model for the ESCRT-mediated ILV formation and degradative sorting at endosomes.

This is a carefully executed study that combines quantitative live imaging and elaborate EM analyses to reveal the dynamics of ESCRT and clathrin action at endosomes in mammalian cells. Given the importance of the ESCRT machinery not only for degradative sorting but also to suppress cancer and facilitate virus budding, this study will be of wide interest to the community. While the descriptive elements of this work are compelling some of the mechanistic aspects require some additional clarification and further experimental support.

1. The relationship between clathrin and Hrs levels on endosomes remains unclear. I miss a firm biochemical proof that the Hrs770 mutant indeed fails to bind to clathrin. Hrs770 puncta in Suppl. Fig. 4D appear much larger than those of WT-Hrs. Does this reflect an increased concentration of Hrs/ endosome or increased endosome size as suggested by Suppl. Fig. 4E?

2. Is the sustained recruitment of Hrs770 still dependent on PI3P? Does manipulation of clathrin binding to Hrs or clathrin itself affect endosomal PI3P levels that could serve to retain Hrs770 on endosomes? Moreover, one would expect that sustained Hrs recruitment to endosomes is also observed upon clathrin knockdown. This can be easily tested.

3. Surprisingly, CHMP4B recruitment kinetics appear to be unperturbed by loss of clathrin binding to Hrs. Together with the sustained presence of Hrs on endosomes the authors speculate that clathrin association is required to trigger Hrs dissociation as ILVs are formed. This model raises a number of important mechanistic questions: Does loss of Hrs affect CHMP4B recruitment? In my (perhaps naive) view, ESCRT-O (e.g. Hrs) serves as a platform for the assembly of ESCRT-I/II complexes onto endosomal membranes. Am I wrong? How does lack of clathrin association with Hrs or loss of clathrin itself affect downstream ESCRT components, e.g. ESCRT-I? Does loss of clathrin binding affect complex formation with ESCRT-I, e.g. via conformational changes that couple clathrin association to some the binding of Hrs to other factors? Such experiments would greatly strengthen the mechanistic aspects of this study.

4. The model proposes that cargo sorting and concentration is mediated by clathrin/ Hrs during the early phase of ILV formation. If so, one would expect that ILV buds formed in Hrs770 expressing cells fail to concentrate cargo. As the authors are expert in immunogold EM labeling this prediction is testable.

Minor points:
5. In Fig. 1 Mander's correlation coefficients are used to quantify colocalization. As the MCC depends on expression level I suggest to re-analyze the data by Pearson's correlation, which should provide more reliable results.

6. CHMP4B KD cells appear to display elevated total EGFR levels, likely as a result of impaired ILV formation and lysosomal degradation. Is the same observed for Hrs770 expressing cells?

7. On p. 7/ bottom the text refers to Fig. 3D when Figure 4D is meant.

8. The rescue data using Hrs-WT or Hrs770 shown in Suppl. Fig. S1F and Suppl. Fig. 4A-D seem to originate from different experiments. Although not mandatory, it would be nice if experiments involving WT and mutant Hrs were performed side-by-side to allow for a direct comparison of the results.

9. What do the asterisks in the blots shown in Suppl. Fig. S1A,B refer to? The nature of the various bands should be described in the legend.

Reviewer #3 (Remarks to the Author):

In this manuscript, Wenzel and collaborators exploiting imaging methods and image analysis investigate in detail the association of ESCRT subunits to the maturing endosomal membrane and the consequent formation of intraluminal vesicles.
The authors use HeLa cells stably expressing GFP constructs to follow such dynamics before and after EGF stimulation.

It is, in it's all, a very interesting and thorough study.

One could question on whether the expression of the GFP constructs of CHMP subunits could interfere with the normal process. The authors do the appropriate controls to address the issue.

-One issue that this reviewer is concerned is on the observations by electron microscopy.
As the authors state, invaginations preceding the formation of ILVs appear close to the clathrin coat. In the manuscript by Sachse et al. (ref 5 in the manuscript) the authors put forward the concept that the invaginations form just adjacent but not really underneath.
Here several micrographs show that the invaginations and ILVs are formed and accumulate firstly really underneath the coat? Like tethered? It is pretty interesting.
Do the ILVs are then only free in the lumen once the coat disassembles?
Also from some micrographs as those in Fig 6 it looks like part of the electron dense coati s engulfed into the bud and vesicle? Which certainly explains the presence of not only Tsg101 but also other subunits and clathrin showed in mass spectrometry analysis of ILVs. This aspect would deserve an additional comment?
- The authors would like certainly to explain to the reader why there is so much EGFR labelling not really associated to the ILVs, a lot of labelling is rather associated with the inner membrane of the maturing MVB. Is there any explanation?

Reviewer #1 (Remarks to the Author):

Review of "Concerted ESCRT and Clathrin recruitment waves define the timing and morphology of intraluminal vesicle formation".

The authors set out to investigate the timing of ESCRT and clathrin recruitment during the formation of intraluminal vesicles involved in receptor signaling degradation. This is an important research topic and the gained insight could help to understand the mechanism of how ESCRTs support scission. However, this reviewer believes that there are some potentially serious problems that need to be addressed in both how the experiments were conducted or interpreted that currently restrain the ability to evaluate significance. In particular, this would be a much strong work if the authors could better characterize the cell lines created for this work, redo certain experiments (as explained further below) and perform additional experiments (suggestions given below).

Major issues:

1. It is claimed that the CHMP4B cell line expresses CHMP4B-GFP at low levels. However, in the Western Blot (Suppl. Figure S1 B) there is much greater than 20-fold overexpression of the GFP-tagged version compared to the endogenous version. All other cell lines generated for this study do not seem to have been characterized (there is no characterization described other than stating that "additional stable HeLa cell lines with close to endogenous expression"), no WBs were shown of the expression levels of the stably expressed proteins, no cell behavior (such as percentage of multinucleated cells or growth rates compared to parental strains) was tested. This is a major problem since it is well established that even modest over-expression of ESCRTs inhibits the processes they are involved in. There are also numerous claims that overexpressed ESCRTs can been found in many non-native locations. Thus, assuming a 20x overexpression (and in the provided Western Blot, it looks greater), even if the CHMP4B-GFP is functional it may be that 5% of what is expressed is sufficient to complement what is lost by the siRNA, and that is properly localized. However, the remaining 95% is mislocalized. The methods section does not give any detail on how these cell lines were generated or tested, other than the authors thanking the FACS facility for help for sorting stable cell lines, so possibly clonal cell lines were established? This is a major concern and needs to be addressed before any of the results gained from experiments with these cell lines can be properly evaluated.

We agree with the reviewer that it is important to characterize the stable cell lines. In the previous version of the manuscript we have indeed characterized the cell lines that we use to draw the main conclusions. We show expression levels of CHMP4B-GFP, mCh-HRSWT and mCh-HRS770 compared to endogenous levels (old Figs. S1A,B,E,F, S4B). We also perform rescue experiments of EGFR degradation and ILV formation using WB, IF and EM, and show that these tagged ESCRT proteins are functional for the process that we are studying in our manuscript (old Figs S1A-F, 5I,J, S4B-F). Further we show that CHMP4B-GFP and mCh-HRS localizes to endosomes in fixed and living cells (old Figs. 1C, 2A, 2C, 3A, S4 etc.), and we never observe class E compartments. In addition, CHMP4B-GFP (not shown) and mEGFP-TSG101 (new Fig. 4H) localize to midbodies. The other cell lines expressing various ESCRT proteins showed similar localizations and/or dynamics as either CHMP4B or HRS and are therefore most likely equally functional.

Moreover, we did not observe differences in speed of proliferation during cultivation of our stable ESCRT expressing cell lines. However, to measure proliferation and multinucleation, we have now performed FACS analyses for DNA content and a mitotic marker. We verified that the expression of tagged ESCRT proteins in

our stable cell lines neither increases the number of bi- or multinucleate cells, nor affects their proliferation or mitotic profile when compared to parental HeLa cells (new Fig. S2). We refer to these additional figures in the text on p. 4. Taken together, we are very convinced that the stable ESCRT expressing cell lines used in this manuscript are functional.

We share the concern by the reviewer that overexpression of ESCRT proteins can preclude the processes they are involved in, and it is therefore important that the stable cell lines express close to endogenous levels. To ensure this, we have exclusively used stably expressing cell lines, either under control of a weakly expressing PGK promotor, or in the case of CHMP3 and CHMP4B under their native promotor (BAC transgenomics). We have now included WBs showing the expression of tagged ESCRT proteins used in this study (new Fig. S1). mCh-HRS, mCh-HD-PTP, mEGFP-TSG101, EGFP-VPS4A and CHMP4B-mCh show equal or less expression compared to endogenous levels. The HeLa-CHMP3-GFP and HeLa-CHMP4B-GFP (BAC) lines were a gift from the Hyman lab and those lines were used and characterized previously by us and others [1-5]. The reviewer is right that the CHMP4B-GFP-BAC line expresses higher than endogenous CHMP4B, based on our old Fig. S1B. We apologize that we chose originally a long exposed WB which made it difficult to compare the intensities, and we have now replaced this WB for a shorter exposed version (new Fig. S3B). In addition we show another WB example of CHMP4B-GFP-BAC (new Fig. S1, lower, right). Quantification of the WB signal shows a level of overexpression of 3.71 +/- 0.15 SD (from 4 WBs) when compared to endogenous CHMP4B. Importantly, as mentioned before, this cell line can fully rescue EGFR degradation, shows a normal cell proliferation pattern and CHMP4B-GFP dynamically localizes to endosomes. We could not compare CHMP3-GFP expression to endogenous levels, due to lack of a working antibody. Importantly, CHMP3-GFP shows similar endosome localization dynamics as CHMP4B and VPS4A.

For the generation of the stable cell lines, the old methods section stated “all other stable cell lines were lentivirus-generated pools, generated as described in [Campeau 2009][6]." Some of the cell lines were sorted by FACS to ensure that we excluded the highest expressing cells from the pools. In addition to referencing to the original publication, we have now included a more detailed description in the methods section.

2. The described limit for co-localization of 5 pixels (400 nm) seems too high. Since the structures that are tested for co-localization are, at least according to the figures shown, much smaller than 400 nm, this reviewer believes that the limit must be set to smaller values. Thus, this reviewer is not convinced that the markers tested do indeed colocalize as stated in the manuscript.

We are unsure which structures the reviewer refers to. From old/new Fig. 2C it should be apparent that the tracked endosomes are approx. 400-500 nm in diameter and that the fluorescently tagged proteins (i.e. HRS and CHMP4B in Fig. 2C) colocalize well with the EGF marker. To clarify this point we include a modified version of old/new Fig. 2C below, where we insert a circle with a diameter of 400 nm to visualize the co-occurrence of two proteins on the same endosome (Fig. A for reviewers). We have in addition measured endosome sizes by EM (old Fig. S4E, new Fig. S7C), confirming that endosomes are approximately 400-500 nm in diameter.

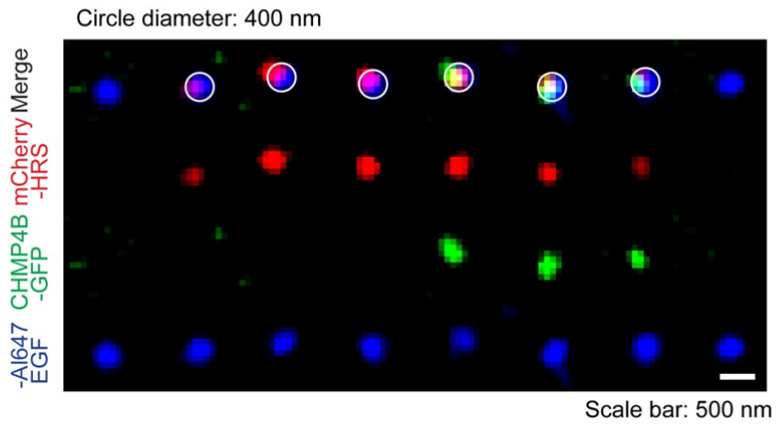

Figure A: Tracking of individual EGF-positive endosomes shows recruitment and dissociation of mCherry-HRS and CHMP4B-GFP. Displayed are individual images of one representative endosome at the indicated time points. The circle of 400 nm visualizes the co-occurrence of two proteins on the same endosome.

We quantify endosomal localization of ESCRT proteins by fixed (old/new Fig. 1) and live-cell imaging (old/new Fig. 2), which give qualitatively similar results over time even though different types of analysis are used: Manders' colocalization coefficients without pre-defined distance (old/new Fig. 1) and object-based colocalization analysis (old/new Fig. 2). However, we agree that the term "colocalization" may be replaced for "co-occurrence" when we talk about the localization of proteins on organelles such as endosomes. We have specified this now in the text and figures.

We have also reanalyzed two datasets with a smaller distance (3 pixels = 240 nm), which is in the same range as our resolution limit. The results look qualitatively very similar, so that we don't see the necessity to re-analyze all datasets with a different threshold (Fig. B for reviewers).

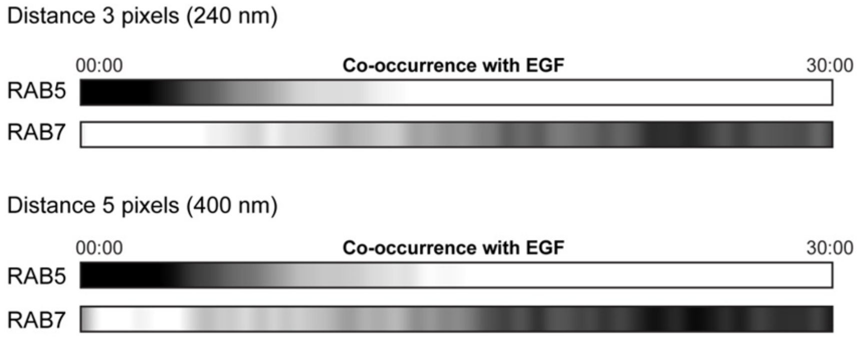

Figure B: Frame-by-frame co-occurrence analysis as explained in old/new Fig. 2B to test whether changing the limit influences the result.

3. The involvement of clathrin in this process is an interesting and potentially important story. Obviously, any disruption of clathrin will affect many steps of membrane transport in the endocytic system and thus affect sorting and targeting in the multivesicular bodies. Thus, a first step is to nail down the correlation of co-localization and then explore if there is a causal link. In Suppl. Figure 4 A to test for co-localization it

needs to be quantified. Additionally, what is the relationship/localization of HRS relative to clathrin in cells not treated with siRNA? This is important to be able to judge the rescue experiments. To this reviewer the image shown for the HRS770 rescue looks as if there are a few spots with co-localization. The evidence for a link would be stronger if this experiment was done with EGF stimulation to test if clathrin is co-localizing to endosomes with the mutant HRS upon stimulation. This would help to establish clathrin as the cause of the ILV phenotype when HRS770 is used. If clathrin still gets recruited, then the given explanation of clathrin causing the failure to form ILVs of the correct size and shape would be wrong. A second problem is that it is not clear which label on the secondary antibody was used to generate these images? Is it compatible with imaging GFP or in this case, making sure that GFP does not contribute to the signal?

As the reviewer points out, we chose a very specific approach to manipulate clathrin recruitment to endosomes by using the HRS770 mutant rescue setups. The HRS770 mutant has been characterized extensively in previous studies from our lab[7,8]. However apparently, we did not refer well enough to these previous publications which characterize the HRS770 mutant in depth, by Y2H, GST pulldowns with in vitro protein, GST pulldowns with cell lysate and by investigating the endosomal localization (IF stainings in transiently overexpressing cells), showing that deletion of the 5 C-terminal amino acids comprising the clathrin box motif of HRS abolishes clathrin binding as well as clathrin recruitment to endosomes.

In the current manuscript, we intended to characterize the HRS770 mutant in an elegant and more physiologic system, namely in cell lines stably expressing endogenous levels of siRNA-resistant wt and mutant HRS, and where the endogenous protein is depleted with siRNA, resulting in a replacement with equal amounts of protein. We agree with the reviewer that it would be helpful to compare endosomal clathrin localization in our stable wt and mutant cell lines in scr versus HRS KD cells and plus/minus EGF stimulation. We have therefore done the suggested IF experiment and also quantify the amount of colocalization. As expected, HRS770 colocalization with clathrin is completely abolished upon KD of endogenous HRS, with or without EGF stimulation (new Fig. S5A). Indeed, in this experiment, the very weak GFP signal from CHMP4B-GFP was not boosted with an anti-GFP antibody and is under these imaging conditions not detectable. We now include the control experiment to prove this point in the manuscript (new Fig. S5B).

In addition, reviewer 2 requested biochemical testing of the HRSwt versus HRS770 interaction with clathrin. We have therefore done Co-IP experiments and also included +/- EGF stimulation in these (new Fig. S5C). Thus, we see the use of the siRNA-resistant HRS770 mutant stable cell lines as a valid tool to study the role of clathrin on endosomes.

4. Please explain the mCherry experiment in Figure 5E, this reviewer cannot find any reference to it.

We thank the reviewer for pointing this out. mCherry is actually not the correct label, since we do not express mCherry in this line, but just measured noise in the red channel. We have therefore removed the measurement in the red channel (new Fig. 5C) and now refer to the figure in the results part on p.8.

5. In general, the results with HRS with deletion of its clathrin-interacting domain (HRS770) are presented in a way as if this proves causality for the failure to generate ILVs. While this is a nice demonstration, this reviewer believes that more experiments are necessary to be able to make this claim. Some are already

described above, but there should also live cell imaging experiments performed in cells where the behavior of clathrin is imaged together with HRS or HRS770 and CHMP4B in cells that were treated with EGF and in non-stimulated cells.

Since we always use EGF-Al647 to label endosomes, imaging of unstimulated cells is technically not possible, and neither is four-colour live-cell imaging possible for us. However, we have now imaged CHMP4B-GFP together with mCh-Clathrin and GFP-HRS770 together with mCh-Clathrin. Trackings from these experiments are included in new Figs. 4A and Suppl. Fig. S5D. Also see comments to point 3.

6. In my opinion, the results do not support the statement on page 8 that "This indicates that clathrin regulates the dissociation of ESCRT-0, but not ESCRT-III.", it was only shown that clathrin is involved in the dissociation of HRS.

We have now further investigated which ESCRT complexes are affected by the absence of endosomal clathrin and find that besides HRS, also TSG101 is hyperstabilized on endosomes upon clathrin depletion (new Fig. 4I, H). In contrast, CHMP4B-GFP showed unperturbed kinetics in the absence of endosomal clathrin (old Fig. 5 E-G, new Figs. 4F and 5C-E). Since ESCRT subcomplexes were shown to be relatively stable and are destabilized as a whole when one subunit is depleted[9,10], we assume that the behaviour of individual subunits reflects the behaviour of the respective subcomplex.

7. Page 10, Phase 1 paragraph, line 9: It is hard to follow why the authors suggest "that clathrin alters the membrane tension required for initiating pit formation." What results is this based on? Clathrin by itself cannot interact with the membrane bilayer, it interacts through various adapters, so how is membrane tension affected? The authors use this argument to explain how absence of clathrin causes the failure to form ILVs, so this is an important and should be explained how the authors believe that this occurs. Related to this, the authors state on page 11, third paragraph, line 4, that "clathrin indirectly regulates" the dissociation of HRS from endosomes upon its phosphorylation, but no mechanism is suggested. Do the authors believe that auxilin and synaptojanin (mentioned further below in the manuscript) are involved in the endosomal setting too? That can be tested via imaging.

Similar to clathrin-mediated endocytosis (CME), clathrin may affect membrane tension via adaptor proteins: Clathrin makes direct contact with HRS and HRS binds both the limiting membrane (via PtdIns3P) and the transmembrane cargo (via ubiquitin). Clathrin may therefore fulfil an important biomechanical role. We have now improved the discussion as suggested by the reviewer (p.11). We are also currently planning a follow up study in collaboration with biophysicists to address these ideas in detail. However given the complexity of this topic, we are not able to resolve these detailed mechanistic questions in the current manuscript.

In addition, in an effort to elucidate the mechanism of how clathrin regulates ILV formation, we have now observed an increase in the levels of endosomal PtdIns3P in cells without clathrin recruitment to endosomes (HRS770 mutant and KD of clathrin) and we report these data now in the manuscript (new Figs. 6H and Suppl. Fig. S6C,D). We further discuss that clathrin may recruit a protein which mediates the turnover of PtdIns3P. Of course we cannot rule out that other molecular mechanisms may contribute to the function of clathrin on endosomes and discuss that it may be either via directly regulating HRS (e.g. via regulating posttranslational modifications of HRS), which may change the affinity of HRS to the endosomal

membrane, or indirectly by recruiting (an)other protein(s) in analogy to CME. We have now removed the names of the proteins involved in clathrin uncoating in CME, since they only served to explain an analogy.

8. The summary paragraph states that the authors have "uncovered novel functions for clathrin in these processes", while this reviewer believes that the authors only showed that clathrin is present, not necessarily that it functions in ILV generation.

Since the reviewer has expressed concerns about the function of the HRS770 mutant above, we understand that the reviewer did not agree with our conclusions about the role of endosomal clathrin in receptor sorting and ILV formation. We hope that our extensive answers to the previous points about the validation of the HRS770 mutant can convince the reviewer that the summary paragraph was not overstated.

In addition, to further strengthen the point that clathrin is important for ILV formation, we now also include clathrin knockdown experiments and observe the same phenotypes as with the clathrin-recruitment deficient HRS770 mutant: Hyperstabilization of HRS on endosomes and prolonged dwell times of HRS (but not CHMP4B) on endosomes (new Fig. 4).

Thus, since we now manipulate the abundance of clathrin on endosomes in two independent ways and observe the same phenotypes, we believe that our statement on p. 14: *"In conclusion, we have established the dynamics and timing of ESCRT recruitment and ILV biogenesis (Fig. 9A) and uncovered novel functions for clathrin in these processes (Fig. 9B)."* is valid.

Minor issues:

1. Details seem to be missing in how the fixed cell experiments have been performed: If live "cells grown on coverslips were permeabilized with PEM buffer", does this mean they were treated with detergent for 10min and then fixed? This raises the concern that a lot of cytosolic signal was lost before fixation possibly invalidating the results shown after such a prolonged permeabilization. The immunofluorescence images might not be representative for the protein distribution in live cells. No detergent was mentioned in the description of the PEM buffer. Can the PEM Buffer permeabilize the membrane or were other chemicals used that are not mentioned in the methods? Some clarification is needed in the methods section.

Yes indeed the PEM buffer contains a mild detergent to permeabilize the plasma membrane before fixation and we apologize that this was not clear. We have now included this in the methods section: "Cells grown on coverslips were permeabilized **with 0.05 % saponin in** PEM buffer...", and added a reference where the buffer and the procedure is described [11]. Saponin extracts cholesterol which is mainly found in the plasma membrane. This procedure, which we use routinely, indeed serves to reduce the cytosolic background of proteins without affecting their endosomal localization.

The following figure (Fig. C for reviewers) illustrates the effect of prepermabilization: The soluble pool of HRS is greatly reduced in the prepermeabilized cells, facilitating analysis of endosomal proteins. Importantly, in live cell imaging, the endosomal signal is still visible over the cytosolic background, enabling tracking of individual endosomes.

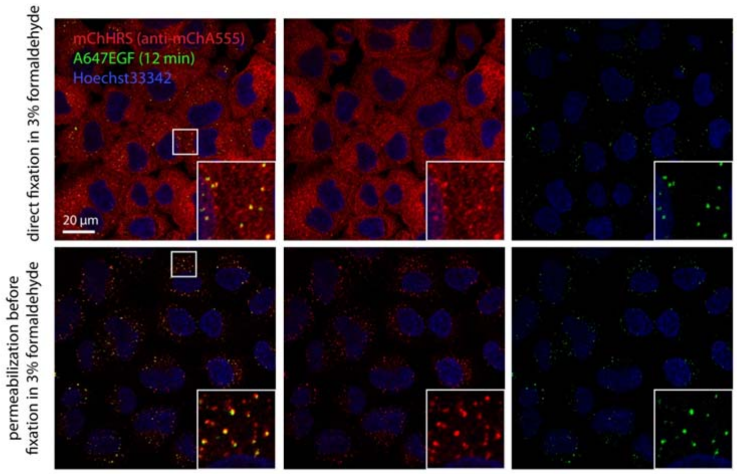

Figure C: Comparison of cells prepermeabilized or not before fixation.

2. In figure 3 D the fluorescence intensity signal seems very high for signal from single endosomes (peaks around 20,000 a.u.) in live-cell imaging over 30 minutes. What laser power was used? At that laser power, what is the time course for bleaching? Was the signal amplified via applying a camera gain? Then this should be described in the methods section.

For conventional widefield fluorescence microscopy, we used the InsightSSI (solid state illumination) light sources (GE healthcare, no laser) at 31% intensity with exposures of 20-40 ms. This usually resulted in raw intensites of below 600 counts, with a camera background between 80-100 counts in a 16-bit image. As we are using sCMOS cameras, there is no additional camera gain applied.

We sometimes observe mild photobleaching. We evaluate for bleaching routinely in the post-processing and correct for bleaching when required (ImageJ bleach correction). We state this now in the methods section. The high AU values result from the deconvolution process, where the original 16-bit image gets transformed into a 32-bit image.

3. Please specify which sCMOS cameras were used. How were the three cameras aligned and how was the registration tested?

Our OMX Blaze V4 is equipped with three PCO.edge sCMOS cameras. The light path uses a fixed dichroic assembly that splits emitted light into the 3 camera light paths. Hardware alignment is done twice a year by GE healthcare service personal and tuned by the core facility personnel if required. Hardware xyz alignments are controlled regularly and are adjusted if necessary by our core facility staff using bead slides. Residual shifts are corrected using a software algorithm that is integrated in the SoftWorx image processing suite. To this end, we measured a dedicated alignment target, the "GE Image Registration slide". This slide consists of a metal film containing a fixed array of sub-diffraction holes which are illuminated using transmitted light and are recorded as an array of diffraction-limited spots. The centers of these spots are identified by fitting, shifts between channels are calculated and an aligned final image is generated by the SoftWorx software using a transformation algorithm.
Detailed information on the process is available under the following address:
https://microscopy.jhmi.edu/Learn/refman/GE/DVOMXSR_ImageAlignment_04-720165-000CC.pdf

To guarantee optimal xy alignment for every experimental setup, we test the alignment before and after every live-cell imaging session by using the "GE Image Registration slide". When required, the alignment file was re-calibrated with the help of this slide before entering image files into post-processing for deconvolution and alignment. This is now described in the methods.

4. Two references in the Methods section of “Immunostaining, antibodies and reagents” were not formatted, the second one is altogether missing in the list of references, the first one could be ref 3 or ref 27.

We have corrected this.

5. The objective used on the Zeiss LSM microscope was misspelled: 631.4NA should be replaced by 63x 1.4 N.A.

We have corrected this.

6. Page 7, last paragraph, sixth line: the wrong figure number was given, it should be 4D instead if 3D.

We have corrected this.

7. Figure 4 D: the y axis has no label
We have corrected this.

8. Suppl. Figures S1: please explain the * and ** on the WBs on the left of A and B.
We have now explained the symbol in the figure legend.

9. The reference for the antibody to Chmp is not formatted properly.
We have corrected this.

Reviewer #2
Wenzel et al in this study have used live imaging to study the dynamics of ESCRT and clathrin recruitment during intralumenal vesicle (ILV) formation at endosomes. Using stable cell lines that mildly overexpress fluorescent protein tagged variants of various ESCRT proteins or clathrin they show that the ESCRT-O component Hrs is gradually recruited over minutes whereas the late-acting ESCRT-III factor CHMP4B displays transient waves of recruitment before it dissociates together with Hrs, likely reflecting the formation of a single ILV as suggested by EM experiments. Interestingly, clathrin, which was shown before to form an Hrs-dependent coat on early endosomes regulates ILV formation and sorting by modulating ILV bud size and by facilitating concerted Hrs removal. These data are taken to propose a new model for the ESCRT-mediated ILV formation and degradative sorting at endosomes.

This is a carefully executed study that combines quantitative live imaging and elaborate EM analyses to reveal the dynamics of ESCRT and clathrin action at endosomes in mammalian cells. Given the importance of the ESCRT machinery not only for degradative sorting but also to suppress cancer and facilitate virus

budding, this study will be of wide interest to the community. While the descriptive elements of this work are compelling some of the mechanistic aspects require some additional clarification and further experimental support.

1.

The relationship between clathrin and Hrs levels on endosomes remains unclear. I miss a firm biochemical proof that the Hrs770 mutant indeed fails to bind to clathrin. Hrs770 puncta in Suppl. Fig. 4D appear much larger than those of WT-Hrs. Does this reflect an increased concentration of Hrs/ endosome or increased endosome size as suggested by Suppl. Fig. 4E?

We apologize that we obviously did not refer well enough to previous publications, which characterized the HRS770 mutant in depth by Y2H, GST pulldowns with in vitro protein, GST pulldowns with cell lysate and by investigating the endosomal localization by IF stainings in transiently overexpressing cells[7,8]. We now refer better to the previous publications and include an extensive quantitative IF experiment as suggested by Reviewer 1 in the presence and absence of EGF stimulation, which shows that the HRS770 mutant fails to recruit clathrin to endosomes (new Fig. S5A,B). In addition, we include a Co-IP experiment as biochemical proof that HRS770 does not interact with clathrin, as suggested by this reviewer (new Fig. S5C).

As the reviewer correctly points out, the endosomes in siHRS/mChHrs770 cells are a bit larger, as also observed in siHRS cells (measured in old FigS4E, new Fig. S7C) and evident from IF (old FigS4D, new Fig. S7B). In addition the mChHRS770 endosomes in old FigS4D, new Fig. S7B appear more intense due to sustained HRS770 localization to endosomes (measured in old Fig5E,F, new Fig. 5C,D). Interestingly, we observe the same in clathrin KD cells and include now quantifications of the increases in endosomal HRS fluorescence intensities upon lack of endosomal clathrin by quantitative high-content microscopy (new Figs. 4D,E, Fig. 6H). Importantly, when we quantified fluorescence intensities of the endosomal protein RAB5 as a control, we did not measure any fluorescence increase of RAB5, confirming the specificity of the hyperrecuitment of HRS upon clathrin manipulation (new Fig. S5E). So the answer is a combination of increased size and increased HRS concentration.

2.

Is the sustained recruitment of Hrs770 still dependent on PI3P? Does manipulation of clathrin binding to Hrs or clathrin itself affect endosomal PI3P levels that could serve to retain Hrs770 on endosomes? Moreover, one would expect that sustained Hrs recruitment to endosomes is also observed upon clathrin knockdown. This can be easily tested.

We thank the reviewer for excellent suggestions! We have now investigated the dependency of HRS770 on PtdIns3P by SAR405 treatment, which specifically inhibits the new synthesis of PtdIns3P. We quantified the reduction of HRSwt versus HRS770 upon 12 min of SAR405 treatment by quantitative high-content microscopy and report a partial reduction in HRS770 levels on endosomes after SAR405 treatment (reduced to 32,7 % compared to DMSO treated control cells, while HRSwt is almost completely reduced to 2,8%, new Fig. 6G). To elucidate which pool of HRS770 is lost, we did live-cell imaging and tracked newly formed EGF-Al647 positive vesicles in SAR405 treated cells. These vesicles do not recruit HRS770 (new Suppl. Fig. S6B), indicating that the initial recruitment of both HRSwt and HRS770 is highly dependent on PtdIns3P. However, once stabilized, HRS770 seems to be less dependent on newly produced PtdIns3P.

To investigate whether endosomal PtdIns3P levels were affected in the HRS770 mutant, we generated double stable cell lines expressing the PtdIns3P probe GFP-2xFYVE together with siRNA-resistant mCherry-tagged HRSwt or HRS770. Knockdown of endogenous HRS again led to an increased intensity of mCherry-HRS770 on endosomes when compared to HRSwt and importantly, also the GFP-2xFYVE probe was clearly enriched on endosomes (new Fig. 6H). As suggested, we also performed this type of experiment in clathrin knockdown cells, giving exactly the same results (new Fig. S6D).

Since the role of clathrin recruitment to endosomes was one of our major interests, we initially avoided to deplete the whole cell for clathrin, as this would affect also other clathrin-dependent processes. We therefore generated stable cell lines expressing the clathrin binding deficient mutant HRS770 and established an elegant rescue approach. According to the reviewer´s request, we have now performed a number of experiments in clathrin knockdown cells, and importantly clathrin depletion by siRNA perfectly mimics the results obtained with the HRS770 mutant rescue setup. We believe that the new data strengthen our conclusion about endosomal clathrin and thank the reviewer for the suggestion. The results are now presented in a separate main figure (new Fig. 4).

3.

Surprisingly, CHMP4B recruitment kinetics appear to be unperturbed by loss of clathrin binding to Hrs. Together with the sustained presence of Hrs on endosomes the authors speculate that clathrin association is required to trigger Hrs dissociation as ILVs are formed. This model raises a number of important mechanistic questions:

Does loss of Hrs affect CHMP4B recruitment? In my (perhaps naive) view, ESCRT-O (e.g. Hrs) serves as a platform for the assembly of ESCRT-I/II complexes onto endosomal membranes. Am I wrong?

How does lack of clathrin association with Hrs or loss of clathrin itself affect downstream ESCRT components, e.g. ESCRT-I?

Does loss of clathrin binding affect complex formation with ESCRT-I, e.g. via conformational changes that couple clathrin association to some the binding of Hrs to other factors? Such experiments would greatly strengthen the mechanistic aspects of this study.

The reviewer is right. In SAR405 treated cells (old Fig. 3B-D, new Fig. 6B-D) both HRS and CHMP4B are lost from endosomes. More specifically, knockdown of HRS abrogates CHMP4B waves (old Fig. 5E, new Fig. 5C). We cannot currently explain why CHMP4B appears unaffected in cells with hyperstabilized HRS coat. However, as suggested by the reviewer, we have now investigated whether components from ESCRT-I would follow HRS or CHMP4B kinetics in clathrin depleted cells. We observed a strong accumulation of TSG101 on endosomes in clathrin depleted cells (new Fig. 4H) and the same was true in HRS770/siHRS cells (data not shown). Tracking of Al647 labelled endosomes revealed further that TSG101 follows similar kinetics as hyperrecruited HRS (new Fig. 4I). From our results it seems that loss of clathrin results in an increased PtdIns3P pool on endosomes, which leads to a hyperstabilization of the ESCRT-0, and -I subcomplexes, but leaves ESCRT-III recruitment unaffected.

4.

The model proposes that cargo sorting and concentration is mediated by clathrin/ Hrs during the early phase of ILV formation. If so, one would expect that ILV buds formed in Hrs770 expressing cells fail to concentrate cargo. As the authors are expert in immunogold EM labeling this prediction is testable.

From previous experience we know that labeling efficiency for EGFR and/or EGF is rather low in these cells, even using the Tokuyasu-method, which has also adverse effects on endosome morphology due to the chemical fixation. We believe therefore that our internalization assay of gold labelled EGFR combined with high-pressure freezing was a better choice to prove our point. We have further corroborated our model by counting ILV buds that display associated gold particles within a distance of 40 nm and estimated that 43,3% of the ILVs in HRSwt and 16,6 % in HRS770, respectively, are associated with gold particles. The numbers stem from images of budding profiles recorded in an unbiased manner, meaning regardless of their association with gold particles. All results are taken from MVEs with gold labelling on the EM section and we include this data now in the methods (Electron microscopy section) and results parts: "In addition, when counting..." on p. 10.

Minor points:

5. In Fig. 1 Mander's correlation coefficients are used to quantify colocalization. As the MCC depends on expression level I suggest to re-analyze the data by Pearson's correlation, which should provide more reliable results.

There are two colocalization analyses described by E. Manders: The Manders' colocalization coefficients (MCC) and the Manders' overlap coefficient (MOC). The latter is a **correlation** coefficient, while the former measures the fraction of one protein that colocalizes with a second protein. We have used Manders' **colocalization** coefficients (MCC) because we were interested in co-occurrence of two proteins on the same structure independent of signal proportionality (i.e. whether ESCRTs are found on endosomes labelled with an endosomal marker), and **not** in a correlation of their intensities. The latter would be measured by Pearson's correlation coefficient and would be relevant when investigating whether two proteins co-distribute in proportion to one another (i.e. whether protein A recruits protein B). In addition, the Pearson's correlation coefficient is highly susceptible to noise and/or variations in fluorescence intensities, and it is only reliable for high correlations[12]. For the aforementioned reasons we believe that we used the most suitable analysis for our data.

6. CHMP4B KD cells appear to display elevated total EGFR levels, likely as a result of impaired ILV formation and lysosomal degradation. Is the same observed for Hrs770 expressing cells?

We have quantified this from WBs and the reviewer is right: Knockdown of CHMP4B in parental cells leads to an increase in EGFR levels. This is almost completely rescued in CHMP4B-GFP expressing cells. Knockdown of HRS has only a marginal effect on steady-state levels of EGFR in parental cells, which is overcompensated in mChHRSwt (slightly reduced steady-state levels of EGFR). This overcompensation is not seen in HRS770 cells, which instead also show a marginal increase in EGFR levels, similar to parental cells.

7. On p. 7/ bottom the text refers to Fig. 3D when Figure 4D is meant.

We have corrected this.

8. The rescue data using Hrs-WT or Hrs770 shown in Suppl. Fig. S1F and Suppl. Fig. S4B seem to originate from different experiments. Although not mandatory, it would be nice if experiments involving WT and

mutant Hrs were performed side-by-side to allow for a direct comparison of the results.

The experiments were performed side-by-side, but split in the figures for didactic reasons. The cover slips for the IF stainings were made during the same experiments as the WBs and the live-cell imaging as parallel readouts from the same KD experiments. We state this now in the methods part and in the figure legend of new Fig. 7A.

9. What do the asterisks in the blots shown in Suppl. Fig. S1A,B refer to? The nature of the various bands should be described in the legend.

We have now explained the symbol in the figure legend.

Reviewer #3
In this manuscript, Wenzel and collaborators exploiting imaging methods and image analysis investigate in detail the association of ESCRT subunits to the maturing endosomal membrane and the consequent formation of intraluminal vesicles.
The authors use HeLa cells stably expressing GFP constructs to follow such dynamics before and after EGF stimulation.

It is, in it's all, a very interesting and thorough study.

One could question on whether the expression of the GFP constructs of CHMP subunits could interfere with the normal process. The authors do the appropriate controls to address the issue.

-One issue that this reviewer is concerned is on the observations by electron microscopy.
As the authors state, invaginations preceding the formation of ILVs appear close to the clathrin coat. In the manuscript by Sachse et al. (ref 5 in the manuscript) the authors put forward the concept that the invaginations form just adjacent but not really underneath.
Here several micrographs show that the invaginations and ILVs are formed and accumulate firstly really underneath the coat? Like tethered? It is pretty interesting.
Do the ILVs are then only free in the lumen once the coat disassembles?

Yes, we think that the ILVs form directly under the coat. We see budding profiles directly under the coat (old Fig. S5A, new Fig. S8A) and the abscised ILVs stay close to the coat (old Fig. 3F, 4, S3A, 5I, new Fig. 3, 6F, 7C, S4D). We would like to point out that in Martin Sachse´s article the Tokuyasu method was used for labeling, leading to a certain difference in endosome preservation. In addition, also in Sachse et al. 2002[13], many ILVs are observed directly under the clathrin coat (Sachse et al. Fig. 5B, 7A), corroborating our results. We don't have an explanation for this observation and can only speculate that there could be i) intraendosomal components, ii) electrostatic interactions, or iii) lipid components responsible for that. We do not observe anything that looks like they are still tethered to the coat/limiting membrane or to each other.

Also from some micrographs as those in Fig 6 it looks like part of the electron dense coati s engulfed into the bud and vesicle? Which certainly explains the presence of not only Tsg101 but also other subunits and clathrin showed in mass spectrometry analysis of ILVs. This aspect would deserve an additional comment?

We agree with the reviewer and added the following sentence into the results section (p. 10): *"Interestingly, we observed in many instances electron dense material inside the forming ILV, which could reflect the presence of clathrin or ESCRT subunits (Fig. 8B, Fig. S8) in line with published results"*[9,14].

- The authors would like certainly to explain to the reader why there is so much EGFR labelling not really associated to the ILVs, a lot of labelling is rather associated with the inner membrane of the maturing MVB. Is there any explanation?

The gold particles close to the inner membrane of the maturing MVE most likely reflect gold-labelled EGFR which is clustered in microdomains on the endosomal limiting membrane. To clarify the topology, we have now made an inset into new Suppl. Fig. S4A. In addition some of the gold particles which appear close to

the inner membrane of the MVE may actually be associated to ILVs, which remained close to the limiting membrane as discussed in detail above. We have also added a zoom in of the MVE in new Fig 6F which shows a nice example of gold labeled EGFR associated with the internalized ILVs. In addition, ILV associated gold particles can be located above or below the section and therefore not visible. An explanation is now provided in the legend to new Suppl. Fig. S4A.

References

1. Guizetti, J. *et al.* Cortical constriction during abscission involves helices of ESCRT-III-dependent filaments. *Science* **331**, 1616-1620, doi:10.1126/science.1201847 (2011).
2. Vietri, M. *et al.* Spastin and ESCRT-III coordinate mitotic spindle disassembly and nuclear envelope sealing. *Nature* **522**, 231-235, doi:10.1038/nature14408 (2015).
3. Christ, L. *et al.* ALIX and ESCRT-I/II function as parallel ESCRT-III recruiters in cytokinetic abscission. *J Cell Biol* **212**, 499-513, doi:10.1083/jcb.201507009 (2016).
4. Mierzwa, B. E. *et al.* Dynamic subunit turnover in ESCRT-III assemblies is regulated by Vps4 to mediate membrane remodelling during cytokinesis. *Nat Cell Biol* **19**, 787-798, doi:10.1038/ncb3559 (2017).
5. Ding, X. *et al.* High-throughput Nuclear Delivery and Rapid Expression of DNA via Mechanical and Electrical Cell-Membrane Disruption. *Nat Biomed Eng* **1**, doi:10.1038/s41551-017-0039 (2017).
6. Campeau, E. *et al.* A versatile viral system for expression and depletion of proteins in mammalian cells. *PloS one* **4**, e6529, doi:10.1371/journal.pone.0006529 (2009).
7. Raiborg, C., Bache, K. G., Mehlum, A., Stang, E. & Stenmark, H. Hrs recruits clathrin to early endosomes. *EMBO J* **20**, 5008-5021, doi:10.1093/emboj/20.17.5008 (2001).
8. Raiborg, C., Wesche, J., Malerod, L. & Stenmark, H. Flat clathrin coats on endosomes mediate degradative protein sorting by scaffolding Hrs in dynamic microdomains. *J Cell Sci* **119**, 2414-2424, doi:10.1242/jcs.02978 (2006).
9. Malerod, L. *et al.* Cargo-dependent degradation of ESCRT-I as a feedback mechanism to modulate endosomal sorting. *Traffic* **12**, 1211-1226, doi:10.1111/j.1600-0854.2011.01220.x (2011).
10. Bache, K. G. *et al.* The growth-regulatory protein HCRP1/hVps37A is a subunit of mammalian ESCRT-I and mediates receptor down-regulation. *Mol Biol Cell* **15**, 4337-4346, doi:10.1091/mbc.E04-03-0250 (2004).
11. Simonsen, A., Bremnes, B., Ronning, E., Aasland, R. & Stenmark, H. Syntaxin-16, a putative Golgi t-SNARE. *European journal of cell biology* **75**, 223-231, doi:10.1016/S0171-9335(98)80116-7 (1998).
12. Bolte, S. & Cordelieres, F. P. A guided tour into subcellular colocalization analysis in light microscopy. *J Microsc* **224**, 213-232, doi:10.1111/j.1365-2818.2006.01706.x (2006).
13. Sachse, M., Urbe, S., Oorschot, V., Strous, G. J. & Klumperman, J. Bilayered clathrin coats on endosomal vacuoles are involved in protein sorting toward lysosomes. *Mol Biol Cell* **13**, 1313-1328, doi:10.1091/mbc.01-10-0525 (2002).
14. Lo Cicero, A. *et al.* Exosomes released by keratinocytes modulate melanocyte pigmentation. *Nature communications* **6**, 7506, doi:10.1038/ncomms8506 (2015).

REVIEWERS' COMMENTS:

Reviewer #1 (Remarks to the Author):

I would like to thank the authors for the comprehensive and responsive revision of their manuscript. I hope that they feel that their manuscript has been strengthened by the review process. I am satisfied with all of their responses.
Sandy Simon

Reviewer #2 (Remarks to the Author):

The authors have done a great job in revising their exciting Ms that I enthusiastically recommend for publication in Nature Communications.
